# Differentially expressed genes in mycorrhized and nodulated roots of common bean are associated with defense, cell wall architecture, N metabolism, and P metabolism

**DOI:** 10.1371/journal.pone.0182328

**Published:** 2017-08-03

**Authors:** Kalpana Nanjareddy, Manoj-Kumar Arthikala, Brenda-Mariana Gómez, Lourdes Blanco, Miguel Lara

**Affiliations:** 1 Ciencias Agrogenómicas, Escuela Nacional de Estudios Superiores Unidad León- Universidad Nacional Autónoma de México (UNAM), León, Guanajuato, México; 2 Instituto de Fisiología Celular, Universidad Nacional Autónoma de México (UNAM), Ciudad Universitaria, Coyoacan, Ciudad de México, México; 3 Instituto de Biología, Universidad Nacional Autónoma de México (UNAM), Ciudad Universitaria, Coyoacan, Ciudad de México, México; Università Politecnica delle Marche, ITALY

## Abstract

Legumes participate in two important endosymbiotic associations, with phosphorus-acquiring arbuscular mycorrhiza (AM, soil fungi) and with nitrogen-fixing bacterial rhizobia. These divergent symbionts share a common symbiotic signal transduction pathway that facilitates the establishment of mycorrhization and nodulation in legumes. However, the unique and shared downstream genes essential for AM and nodule development have not been identified in crop legumes. Here, we used ion torrent next-generation sequencing to perform comparative transcriptomics of common bean (*Phaseolus vulgaris*) roots colonized by AM or rhizobia. We analyzed global gene expression profiles to identify unique and shared differentially expressed genes (DEGs) that regulate these two symbiotic interactions, and quantitatively compared DEG profiles. We identified 3,219 (1,959 upregulated and 1,260 downregulated) and 2,645 (1,247 upregulated and 1,398 downregulated) unigenes that were differentially expressed in response to mycorrhizal or rhizobial colonization, respectively, compared with uninoculated roots. We obtained quantitative expression profiles of unique and shared genes involved in processes related to defense, cell wall structure, N metabolism, and P metabolism in mycorrhized and nodulated roots. KEGG pathway analysis indicated that most genes involved in jasmonic acid and salicylic acid signaling, N metabolism, and inositol phosphate metabolism are variably expressed during symbiotic interactions. These combined data provide valuable information on symbiotic gene signaling networks that respond to mycorrhizal and rhizobial colonization, and serve as a guide for future genetic strategies to enhance P uptake and N-fixing capacity to increase the net yield of this valuable grain legume.

## Introduction

Legumes have the unique capacity to form symbiotic associations with ancient phosphorus-acquiring arbuscular mycorrhizal fungi (AMF, which arose ~450 million years ago) and recent nitrogen-fixing *Rhizobium* bacteria (which arose ~60 million years ago) in a tripartite relationship [1–4]. AMF belonging to the phylum Glomeromycota help their host plants absorb and translocate the major mineral nutrients phosphorus (P) and nitrogen (N) and minor micronutrients from the soil [5]. Nitrogen-fixing rhizobial bacteria convert atmospheric free nitrogen to ammonia, which the host plant uses for growth and development [6]. In return, the host plant provides the micro-symbionts with photosynthetically fixed carbon compounds [7]. The symbiotic interaction is initiated by a molecular dialogue between the host and AMF or rhizobial partners, which functions to establish a mutually beneficial relationship without invoking host plant defenses. These initial interactions include complex signal perception and transduction networks governed by regulatory genes in both symbiotic partners. In response to plant-derived bioactive signals such as strigolactones [8] and (iso)flavonoids, AMF and rhizobia synthesize and secrete Myc (lipochitooligosaccharide (LCO) [9] and short-chain chitin oligomer (COS) [10]) and Nod (LCO [11]) factors, respectively. The Myc and Nod factors stimulate signaling by a common symbiosis pathway (CSP), which is shared between AMF and rhizobial symbioses [12].

Plant genes and proteins involved in the initial symbiotic signaling interactions were identified and temporally ordered by performing systematic phenotypic analyses of model legume (*Lotus japonicus* and *Medicago truncatula*) mutants arrested at different stages of the symbiotic response [13,14]. The CSP genes such as SYMRK, CASTOR, POLLUX, NUP85, and NUP133 are required for the induction of calcium spiking, a distinctive physiological response during early symbiotic interactions. CALCIUM- AND CALMODULIN-DEPENDENT PROTEIN KINASE (CCAMK, a decoder of calcium signaling) and CYCLOPS are downstream of calcium spiking [12]. In a downstream or parallel event, the GRAS protein transcription factors NSP1, NSP2 [15,16], and RAM1 [17] form complexes that drive symbiont-specific gene expression in legumes. For example, RAM1 and RAM2 induce the biosynthesis of cutin monomers, which are involved in appressorium formation by AMF [18]. NSP1 and NSP2 are sufficient to activate the nodulation-specific ERF transcription factor ERN1 [19] and the downstream transcription factors NIN, NF-YA, and NF-YB, which are involved in the initiation of nodule organogenesis [20,21] along with cytokinin signaling factors [22,23]. Subsequently, the host plant enables colonization by either AMF or rhizobia, depending on its perception of early bioactive signals. Nevertheless, for the successful colonization of these symbionts the legume plants must avoid eliciting defense responses and compromise for cell wall degradation during invasion [24]. Indeed, a transient induction of few defense markers show at early stages of symbionts interactions suggesting that general microbe-associated molecular patterns from AM fungi or rhizobia are perceived and elicit an ephemeral defense response, which later is suppressed [24]. Proteins involved in the blocking this defense pathway and cell wall loosening associated enzymes are poorly understood.

Common bean (*Phaseolus vulgaris*) is the most important crop grain legume and is a rich source of dietary protein. *P*. *vulgaris* participates in both AMF and root nodule symbiosis. RNA interference (RNAi) studies have functionally validated several genes associated with nodule symbiosis in *P*. *vulgaris*, such as the heterotrimeric nuclear factor-YC1 (PvNF-YC1) [25], PvNF-YB [21], PvSYMRK [26], PvRACK1 [27], PvNADPH-OXIDASE [28], PvTRE1 [29], PvNODULIN 22 [30], PvSIN1 [31], and PvTOR [32]. The functions of most of these genes have not been determined in AMF symbiotic interactions. Several recent studies described the functions of *Pv*RBOHB [33,34] and an autophagy-related kinase [35] during AMF symbiosis. However, plant genes that participate in AMF symbiosis are still largely unidentified. Therefore, further research is required to investigate and identify novel genes associated with AMF and root nodule symbioses in common bean. High-throughput technologies such as microarray analysis and next-generation sequencing have been used to examine differential gene expression patterns under AMF and rhizobial symbiotic conditions by performing comparative transcriptome profiling in model legumes [36,37,38,39]. However, these analyses have not been performed in important crop grain legumes. Comparative transcriptome analyses were performed previously using cDNA macroarrays in *L*. *japonicas* [36] and microarrays in *Casuarina glauca* and *M*. *truncatula* symbiotic roots [37]. Rapid advances in RNA-sequencing (RNA-Seq) and associated bioinformatics strategies have provided revolutionary tools for global transcriptomic research on plants [40]. For example, these tools have been used to elucidate gibberellin biosynthesis gene expression patterns [41] and transcription factors [42] associated with mycorrhizal colonization.

In the present study, we analyzed transcriptome changes in *P*. *vulgaris* roots under AMF and nodulated conditions using RNA-Seq technology. Our root transcriptional analysis identified 3,219 and 2,645 genes that were differentially expressed in response to mycorrhizal or rhizobial colonization, respectively. These data were used to predict key modules that control these two divergent root symbionts such as defense response, cell wall integrity, N metabolism and P metabolism. Further, we performed a comparative and quantitative analyses of host gene expression profiles associated with defense, cell wall structure, N metabolism and P metabolism.

## Materials and methods

### Plant materials, symbiont inoculation, and growth conditions

*Phaseolus vulgaris* L. cv. Negro Jamapa seeds obtained from Instituto de Biotecnología, UNAM, Mexico were used for all experiments. Seeds were surface-sterilized by immersion in absolute ethanol for 1 min and 10% sodium hypochlorite for 10 min, followed by three washes with sterile distilled water. Then, seeds were germinated on sterile filter paper moistened with B&D solution [43] in darkness for 2 days at 28°C. Two-day-old germinated seeds were planted in pots containing sterile vermiculite, and maintained under greenhouse conditions with a 16-h photoperiod and 65% relative humidity at 27±1°C. Five-day-old seedlings (n = 9 for each set) were inoculated in the root zone with 1 ml of *Rhizophagus irregularis* spores (1,000 spores/seedling) [Symplanta®, Germany], or *Rhizobium tropici* strain CIAT899 (1 ml/seedling at OD_600_ = 0.05) dx.doi.org/10.17504/protocols.io.h83b9yn. The choice of *R*. *irregularis* and *R*. *tropici* for this study is both are the most studied symbionts and these species are tolerant of stress conditions such as high temperature and acidity [44,45]. Inoculated seedlings were irrigated two times per week with modified B&D solution to promote mycorrhizal colonization (supplemented with 10 μM potassium phosphate [46]) or to promote nodulation (B&D solution without nitrate [47]). A set (n = 9) of uninoculated plants grown separately under identical conditions was used as the control (S1 Fig). At different time points (1, 2, 3 wpi), root samples (3 plants from each set, approximately 3 g) were excised from these plants (*R*. *irregularis*, *R*. *tropici*, and control), and half of the sample was immediately frozen in liquid nitrogen and stored at -80°C for RNA extraction. The remaining half of the sample was used for further analyses such as, calculation of %RLC and quantification phosphorous in mycorrhized and nitrogenase assay in rhizobia inoculated roots, RT-qPCR to confirm the symbiosis and validate the RNA-Seq data.

Based on the PvPT4 expression in AMF inoculated and nitrogen fixing ability and absence of senescenced nodules in rhizobia inoculated roots, 2 wpi roots were selected for transcriptome analysis.

### Assessment of mycorrhization and nodulation

To determine the status of mycorrhizal colonization, the remaining mycorrhized root samples were stained using a modified trypan blue histochemical staining method. Stained root samples were examined under a light microscope (Leica, DMLB bright-field microscope) to visualize fungal structures and determine the percentage of root length colonization (%RLC) according to McGonigle et al. [48]. Total phosphorus concentration in dried leaves was measured using the nitric-perchloric acid method following the protocol described by Miller [49]. The acid-digested samples were dissolved in distilled water and quantified spectrophotometrically. A standard curve was prepared using KH_2_PO_4_. In parallel, ethylene production in nodulated root samples was quantified by subjecting samples to the acetylene reduction assay described previously by Ramírez et al. [50]. The samples were incubated in acetylene gas for 30 min, and ethylene production was determined by gas chromatography (Variant model 3300). Ethylene specific activity was expressed as μl mol^-1^ C_2_H_2_ h^-1^ g^-1^ of nodule dry weight (DW).

### Quantitative real-time PCR analysis

The RT-qPCR analysis was performed to verify the absence of mycorrhiza and rhizobia cross contamination; and also to validate the RNA-Seq data (S1 Table). Six genes each for mycorrhized and nodulated conditions were selected to validate the RNA-Seq data among which, two genes were symbiosis specific, two genes that were highly upregulated and two genes that were highly downregulated. High-quality total RNA was isolated from frozen root tissues using TRIzol reagent (Sigma) according to the manufacturer’s instructions. RNA integrity was verified by gel electrophoresis, and RNA concentration was assessed using a NanoDrop spectrophotometer (Thermo Scientific). DNA contamination was eliminated using RNase-free DNase (1 U/μl; Roche, USA) according to the manufacturer’s instructions. Reverse-transcription quantitative PCR (RT-qPCR) analysis was performed using a DNA-free RNA and iScript^TM^ One-Step RT-PCR Kit with SYBR^®^ Green (Bio-Rad) according to the manufacturer’s instructions. A control sample lacking reverse transcriptase was included to confirm the absence of contaminant DNA. Relative expression values were calculated using the 2^-ΔCt^ method, where the quantification cycle (Cq) value equals the Cq value of the gene of interest minus the Cq value of the reference gene [51]. Gene-specific primers were used for RT-qPCR analysis (S1 Table). The *Phaseolus vulgaris* genes EF1α and IDE were used as reference as described previously by Arthikala et al. [33]. The relative expression values were normalized with respect to two reference genes EF1α and IDE as described previously by Vandesompele et al. [52]. The presented values are averages of three biological replicates, and each data set was recorded using triplicate samples.

### Transcriptome analysis

#### mRNA enrichment

For transcriptome profiling, high-quality total RNA was isolated from frozen root tissues from two biological replicates using the RNeasy® Plant Mini Kit according to the manufacturer’s instructions (Qiagen, Hilden, Germany). Genomic DNA contamination was eliminated by incubating the samples with RNase-free DNase (1 U μl^-1^) at 37°C for 15 min, and then at 65°C for 10 min. RNA quality was tested by measuring the A_260_/A_280_ and A_260_/A_230_ ratios using a NanoDrop Spectrophotometer. Electropherograms were obtained using an Agilent 2100 Bioanalyzer platform (Agilent Technologies, USA) with an Agilent RNA 6000 Nano Kit; Agilent 2100 Expert software version B.02.03.SI307 was used to calculate the RNA integrity number (RIN) [53]. RIN values of samples ranged from 7.0 to 7.5. A 1 μg aliquot of total RNA was subjected to mRNA enrichment using the Dynabeads® mRNA DIRECT™ Micro Kit (Cat. nr. 61021, Life Technologies) according to the manufacturer’s instructions.

#### Library preparation

The cDNA libraries were constructed using the Ion Total RNA-Seq Kit v2 (Cat. nr. 4479789, Life Technologies) according to the manufacturer’s instructions. Briefly, 100 ng of enriched mRNA was fragmented for 10 min with RNase III. Fragmented RNA was purified using nucleic acid binding beads and binding buffers according to the manufacturer’s instructions (Cat. nr. 4475486, Life Technologies). Purified samples were evaluated on an Agilent 2100 Bioanalyzer to assess yield and mRNA fragment size distribution. Then, 25–50 ng of fragmented mRNA was hybridized with ion adapters in a thermocycler for 10 min at 65°C and 5 min at 30°C. Hybridized fragmented mRNA was incubated with ligase (Cat. nr. AM2141, Life Technologies) for 30 min at 30°C to ligate the adapters. Then, the hybridized samples were mixed with reverse transcriptase master mix, and incubated at 42°C for 30 min to generate cDNA libraries. The cDNA libraries were purified using nucleic acid binding beads and buffers according to the manufacturer’s standardized protocol (Cat. nr. 4475486, Life Technologies). The purified cDNA libraries were subjected to PCR amplification using Platinum PCR Supermix High Fidelity and Ion Xpress Barcode reverse and forward primers (Thermo Fisher Scientific) with the following conditions: 95°C for 2 min; two cycles of 94°C for 30 s, 50°C for 30 s, and 68°C for 30 s; 14 cycles of 94°C for 30 s, 62°C for 30 s, and 68°C for 30 s; and a final extension at 68°C for 5 min. The amplified cDNA libraries were purified using nucleic acid binding beads and binding buffers. The yield and size distribution of each library were evaluated using the Agilent 2100 Bioanalyzer.

#### Template preparation, enrichment, and sequencing

Templates were prepared from 10 pM of pooled barcoded cDNA libraries using the Ion PI Template OT2 Solutions 200 Kit v3 (Cat. nr. 4488318, Life Technologies) according to the manufacturer’s instructions. Briefly, 10 pM of pooled cDNA libraries was mixed with Ion PI reagent mix TL, Ion PI PCR reagent B, Ion PI enzyme mix TL, and Ion PI Ion sphere particles v3. The mixtures were vortexed, transferred into an Ion PI Plus reaction filter assembly, and then the assemblies were loaded in the Ion OneTouch 2 instrument for template amplification (Life Technologies). The instrument was turned on and the reactions ran for 6.5 h. Then, the beads were isolated and quality was assessed on a QuBit instrument to determine the percent of beads that contained polyclonal templates. After polyclonal assessment, the samples were enriched using the reagents in the Ion PI Template OT2 Solutions 200 Kit v3 (Cat. nr. 4488318, Life Technologies) and the Ion OneTouch ES instrument, according to the manufacturer’s instructions. After enrichment, the beads were washed and prepared for sequencing. Then, the beads were loaded onto a prepared and calibrated Ion P1 Chip (Life Technologies) according to the manufacturer’s Ion P1 Sequencing 200 Kit v3 protocol. The loaded chip was placed into the Ion Proton Sequencer, and the run was started using an Ion Torrent RNA-Seq run plan that was configured based on the library type, species, required number of run flows, required type of plug-in, adapter trimming, and other parameters that were specific for the RNA-Seq run. RNA sequencing and analysis were performed by the PrimBio Research Institute LLC, Exton, PA, USA.

#### Alignment and data analysis

After the proton run was completed, the raw sequences were aligned to *R*. *irregularis / R*. *tropici* database to eliminate the transcripts aligned to the symbiont’s database (http://fungi.ensembl.org/Rhizophagus_irregularis_daom_197198w/Info/Index, http://genome.annotation.jp/RhizoBase). Next, the filtered sequences were aligned to the *P*. *vulgaris* reference sequence (*Phaseolus vulgaris* v2.1, DOE-JGI and USDA-NIFA, http://phytozome.jgi.doe.gov/) using Strand NGS software version 2.0 (San Francisco, CA). The sequence alignment/map (SAM) files were used for further analysis. Quality control was assessed by the Strand NGS program, which determined the pre- and post-alignment quality of the reads for each sample. The aligned reads were then filtered based on alignment score, match count, mapping quality, and average base quality. After filtering, the aligned reads were normalized and quantified using the differential expression sequences (DEseq) algorithm and the Strand NGS program. Fold change (≥ 2) was determined for each sample by the Strand NGS software based on the normalized data. Gene ontology (GO) analysis was performed on genes that were either upregulated or downregulated for each condition using AgriGO [54] (S1 Fig) and the Classification Super Viewer Tool at BAR (http://bar.utoronto.ca).

Hierarchical clustering was performed according to Eisen et al. [55], and the results were visualized with TreeView. Graph-based visualization of GO categories and interactive graphs were developed by REVIGO [56]. The pathway networks were obtained from KEGG Plant (http://www.kegg.jp/kegg/genome/plant.html) [57]. Heat maps were drawn with fold-change values using the R package (https://www.r-project.org/). Venn diagrams were constructed with DEG numbers using Lucidchart software (https://www.lucidchart.com/).

## Results

### Establishment of *P*. *vulgaris* root symbiosis with AMF and rhizobia

**Fig 1:**
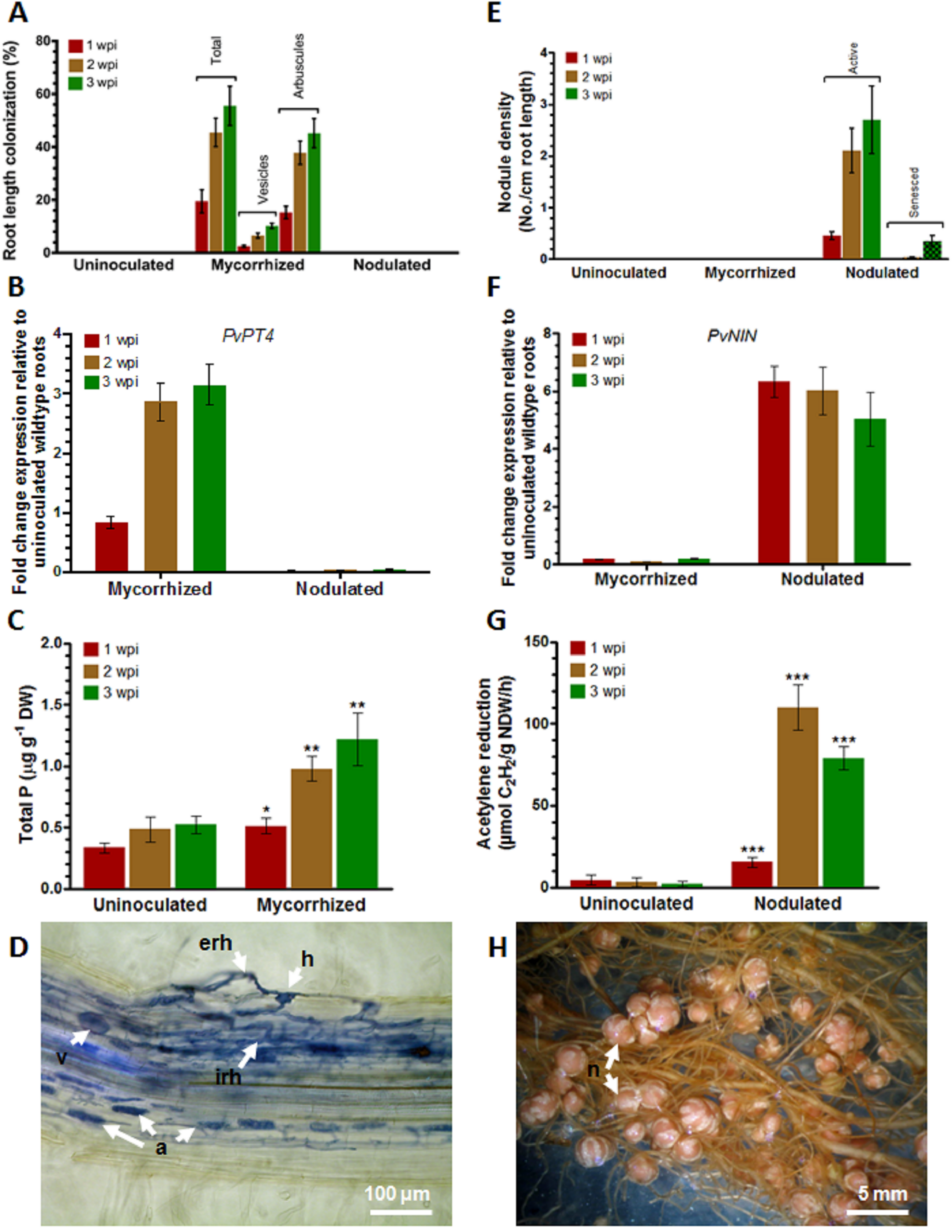
Determination of mycorrhizal and rhizobial colonization in *P*. *vulgaris* roots. Control (uninoculated), mycorrhized (*R*. *irregularis*), and nodulated (*R*. *tropici*) root samples were assessed at different time points for symbiotic colonization. (A) Percent mycorrhizal root length colonization. (B) RT-qPCR analysis of mycorrhizal-induced PvPT4 transcript levels. (C) Total shoot phosphorus concentration. (D) Representative image showing fungal structures in trypan blue-stained roots at 2 weeks post inoculation (wpi) with *R*. *irregularis*. (E) Nodule density. (F) RT-qPCR analysis of rhizobial-induced PvNIN transcript levels. (G) Nitrogen-fixing activity measured by acetylene reduction assay. (H) Representative image showing root nodules at 2 wpi with *R*. *tropici*. erh, extraradical hyphae; h, hyphopodia; irh, intraradical hyphae, v, vesicle; a, arbuscule; n, nodule. Error bars on the graphs represent mean ± SD of three biological replicates (n = 9). Statistically significant differences are indicated by asterisks (unpaired two-tailed Student´s *t* test; *P<0.05, **P<0.01, ***P<0.001).

To elucidate the symbioses-root transcriptome datasets of *P*. *vulgaris*-AMF and *P*. *vulgaris*-rhizobia, root tissues individually colonized either with *R*. *irregularis* or *R*. *tropici* were established. Initially, the colonization kinetics of mycorrhized roots were assessed at three different time points. At 1 week post inoculation (wpi), the mycorrhized wild-type roots contained all relevant fungal structures, including hyphopodia, extraradical hyphae, intraradical hyphae, vesicles, developing (immature) arbuscules, and mature arbuscules. However, at 2 and 3 wpi, the %RLC of total vesicles and arbuscules displayed a >1-fold increase compared with those at 1 wpi (Fig 1A). RT-qPCR analysis was performed for PvPT4 (*P*. *vulgaris PHOSPHATE TRANSPORTER 4*) and PvNIN (*P*. *vulagris NODULE INSEPTION*) to rule out cross contamination of the symbionts due to their specific induction only in presence of mycorrhiza or rhizobia, respectively. RT-qPCR analysis revealed that higher levels (>2-fold) of AMF induced PvPT4 transcript accumulation at 2 wpi, and the same expression levels were maintained at 3 wpi in mycorrhized roots (Fig 1B). Subsequent analysis confirmed that these mycorrhized root samples lacked nodules and rhizobial symbiosis-specific PvNIN transcript, indicating that the samples were free of rhizobial cross-contamination (Figs 1E and 1F). The uninoculated control (henceforth designated as control) root tissues lacked AMF fungal structures at all observed time points (Fig 1A). The total P concentrations were significantly higher in leaves of mycorrhized plants than in control leaves (Fig 1C). Among the leaves of mycorrhized plants P concentrations were found to be increase over one-fold in 2 and 3 wpi samples compared with that at 1 wpi (Fig 1C).

Rhizobial colonization was assessed at three stages (1, 2, and 3 wpi). Similar to mycorrhized roots, rhizobial-inoculated roots display a >3-fold increase in nodule density at 2 and 3 wpi compared with those at 1 wpi. However, roots at 2 wpi lacked senesced nodules and fixed more atmospheric nitrogen than did roots at 1 and 3 wpi (Fig 1E and 1G). Nodulation-specific PvNIN transcript expression was observed only in rhizobial-inoculated roots (Fig 1F). Subsequent analysis confirmed that these nodulated root samples lacked AMF symbiosis-specific PvPT4 transcript expression, and the %RLC was zero, indicating that the samples were free of AMF cross-contamination (Fig 1A and 1B). Control roots also were free of rhizobial contamination (Fig 1E and 1G). These combined results indicate that 2 wpi *P*. *vulgaris* root tissues inoculated with either *R*. *irregularis* (Fig 1D) or *R*. *tropici* (Fig 1H) were ideal for further analysis because they had active P uptake (determined by PvPT4 expression and total leaf P concentrations) and active biological nitrogen fixation.

### Transcriptome profiling and DEGs of *P*. *vulgaris* roots during symbiotic colonization

**Fig 2:**
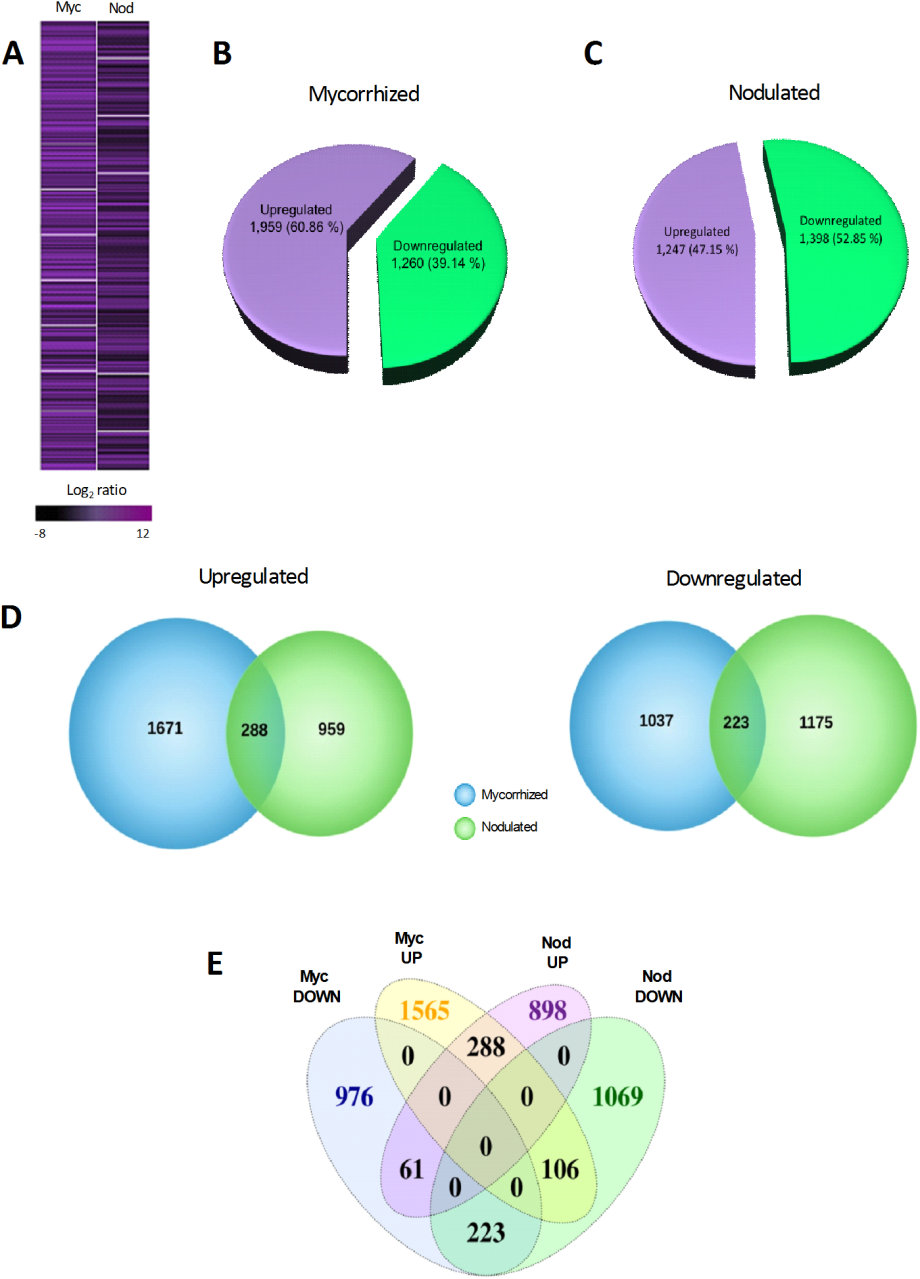
Summary of genome-wide expression profiling and identified DEGs in response to mycorrhizal and rhizobial colonization in *P*. *vulgaris* roots. Global transcriptome profile of genes upregulated and downregulated in response to arbuscular mycorrhizal fungi and rhizobia (unpaired Student´s *t-*test, *P*-values of ≤0.05 and fold-change of ≥2.0 upregulated and downregulated) (A). Number and percent distribution of uniquely expressed upregulated and downregulated genes during (B) mycorrhizal and (C) nodulation conditions. The *P*. *vulgaris* locus names and descriptions are listed in S3 Table. Venn diagram showing (D) shared DEGs (left side, upregulated genes; right side, downregulated genes) and (E) number of overlapping DEGs in upregulated and downregulated mycorrhized and nodulated roots (clustered into four comparison groups represented by four ellipses).

To better understand the molecular landscape of *P*. *vulgaris* genes that respond during colonization of two divergent root symbionts, we performed genome-wide expression profiling of mRNA from control and 2 wpi mycorrhized and nodulated roots using Ion Proton sequencing (S2 Table). Hierarchical cluster-derived control, mycorrhized, and nodulated dendrograms revealed that the transcript expression levels from two independent (biological) replicates of each group clustered together (S2 Fig). These data were then deposited in the National Center for Biotechnology Information (NCBI) with accession number of PRJNA388751 (https://www.ncbi.nlm.nih.gov/bioproject/388751). Based on statistical analysis of expression levels (unpaired Student´s t-test, *P*-values of ≤ 0.05 and fold-change cut-off for upregulation and downregulation of ≥2.0; S3 Table), we identified 1,959 upregulated and 1,260 downregulated *P*. *vulgaris* DEGs during mycorrhizal colonization (Fig 2A and 2B). During nodulation, we identified 1,247 upregulated and 1,398 downregulated DEGs (Fig 2A and 2C). Venn diagrams illustrate the presence of shared DEGs between the two symbiont–plant interactions (Fig 2D). Our analysis revealed that 288 of the total upregulated genes (14.7 and 23% of upregulated genes in mycorrhized and nodulated roots, respectively) and 223 of the total downregulated genes (17.7 and 16% of downregulated genes in mycorrhized and nodulated roots, respectively) overlapped between these symbiotic conditions. The overlapping DEGs clustered into four comparison groups, revealing that 106 genes specifically upregulated during mycorrhization intersect with DEGs that were downregulated during nodulation. Conversely, 61 DEGs that were upregulated during nodulation intersected with DEGs that were downregulated during mycorrhization (Fig 2E).

**Fig 3:**
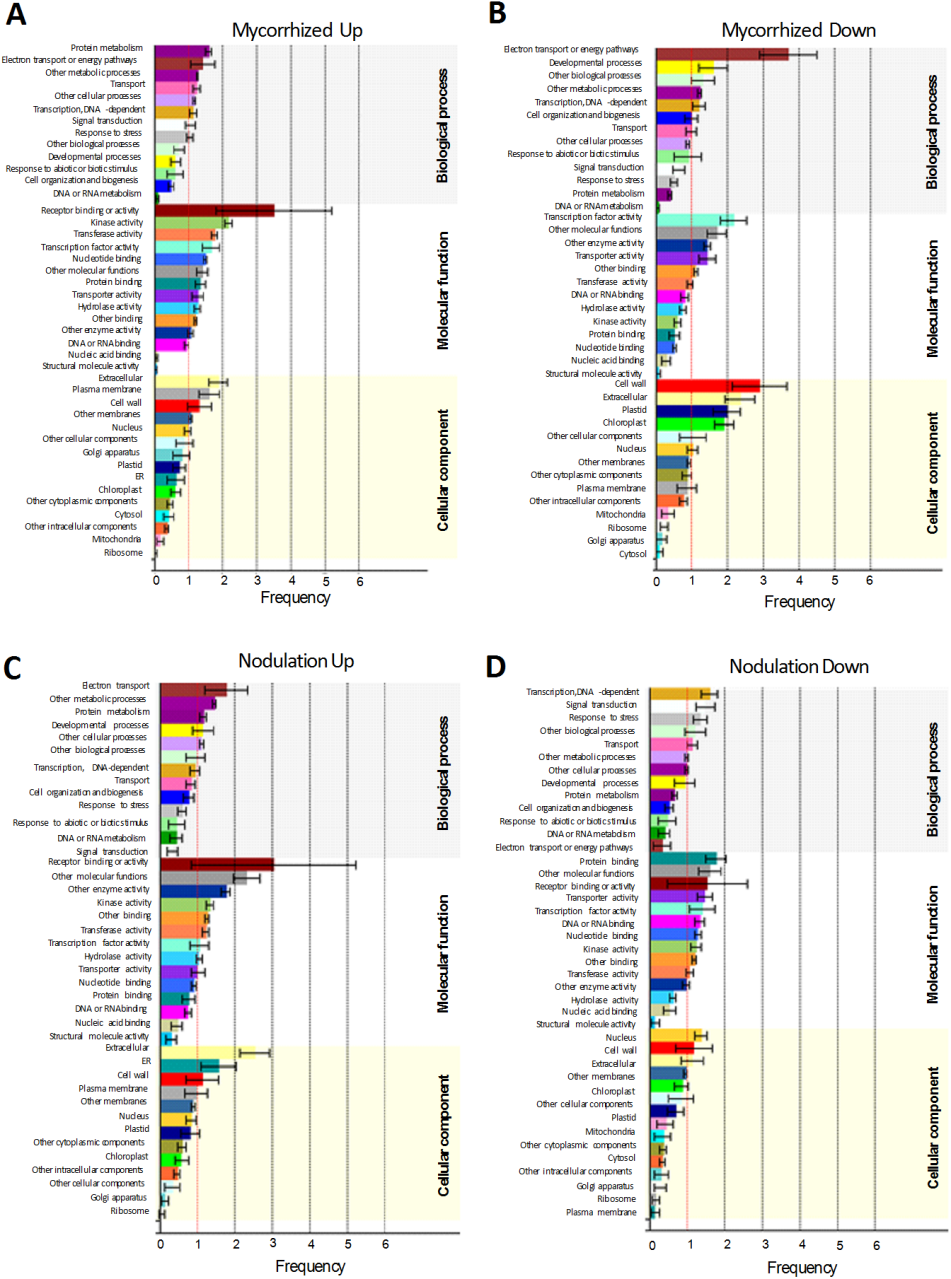
Gene ontology (GO) annotations of differentially expressed genes (DEG) in symbiont-colonized *P*. *vulgaris* roots. GO term annotation of (A) upregulated and (B) downregulated DEGs in mycorrhized roots. GO term annotation of (C) upregulated and (D) downregulated DEGs in nodulated roots. The DEGs were summarized in three main GO categories, biological process, molecular function and cellular component. The analysis was performed using classification Super Viewer tool at The Bio-Analytic Resource (BAR) for Plant Biology (http://bar.utoronto.ca) and the values of normed frequency was used to plot the graphs. The y-axis indicates the sub-categories and the x-axis indicates the frequency of a sub-category of genes in that category.

A total of 1,959 upregulated and 1,260 downregulated mycorrhized DEGs and 1,247 upregulated and 1,398 downregulated nodulated DEGs were assigned to three GO categories, including biological process, molecular function, and cellular component. In the upregulated DEGs of mycorrhized root samples, most of the 47% GO annotations belonged to BP, followed by 39% GO annotations in MF and 14% in CC. In the downregulated mycorrhized root samples 46% belonged to BP, 34% in MF and 20% in CC. Similarly, the total number of GO terms for upregulated nodulated root samples was 35% in BP, 35% in MF, and 30% in CC; in downregulated DEGs of nodulated roots show 46% GO annotations in BP, 34% in MF and 20% in CC (S4 Table). The BP category from both mycorrhized and nodulated roots contained high percent frequency of protein metabolism processes, energy pathways, other metabolic processes, signal transduction, transport and response to stress (Fig 3); minor groups within this category included immune system process, peptide transport, defense response, response to oxidative stress, and cell wall macromolecule catabolism (S4 Table). The most abundant groups in the molecular function category were binding, catalytic activity, hydrolase activity, oxidoreductase activity, kinase (protein) activity, and transferase activity. Minor sub-groups in the molecular function category included transmembrane/transporter activity, transcription factors, peroxidase activity, heme binding, and antioxidant activity. In the CC category most abundant groups were cell wall and plasma membrane in both mycorrhized and nodulated samples. In general, the GO terms remain largely same in all the three categories however, the percent frequency of these GO terms were varied among them (Fig 3A–3D). Next, we used REVIGO to develop a graph-based visualization tool for the biological process and molecular function GO categories (S3 Fig). In mycorrhized root samples, metabolism (phosphorus metabolism, phosphate/nitrogen-containing compound metabolism, glucan metabolism), membrane transport, peroxidase activity, cell wall, oxido-reduction processes and defense-related GO terms were linked together (S3A and S3B Fig). In nodulated root samples, GO terms related to transmembrane transport, binding, peroxidase activity, cell wall, defense, and oxido-reduction processes were linked together (S3C and S3D Fig). The results obtained from GO functional annotations and interactive graphs of the DEGs suggest that several major and minor groups of all three GO categories were present at different percentages in both mycorrhized and nodulated roots of *P*. *vulgaris* (Fig 3; S6 Table).

**Fig 4:**
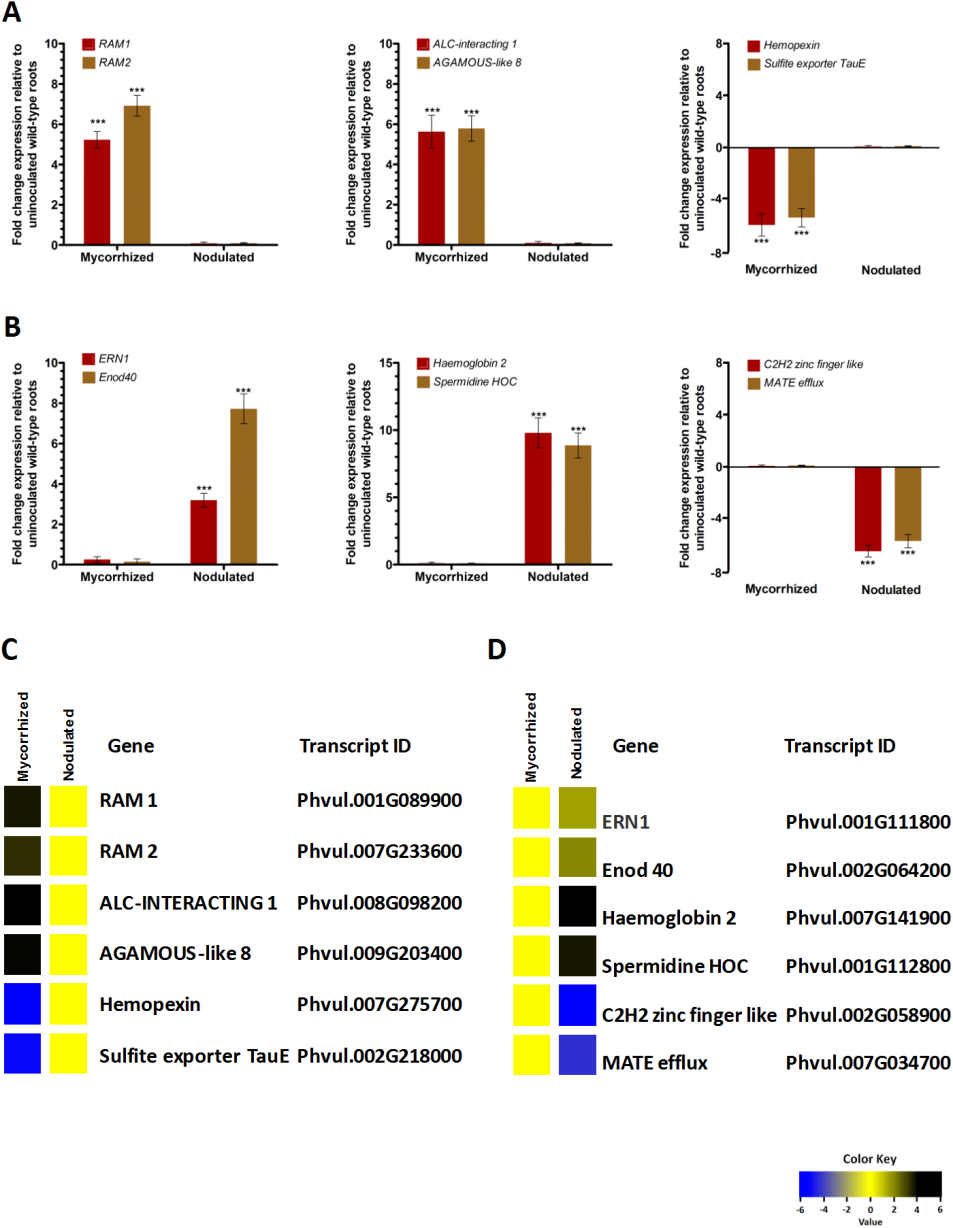
Validation of expression patterns of 12 DEGs of symbiont-colonized *P*. *vulgaris* roots by RT-qPCR analysis. Twelve DEGs (differentially expressed genes) showed similar expression patterns between RT-qPCR data (A and B) and RNA-Seq data (C and D). These genes included four genes well characterized mycorrhizal symbiosis specific RAM1 and RAM2, nodule specific ERN1 and ENOD40; Four genes ALC-INTERACTING 1, AGAMOUS-LIKE 8 and HEMOPEXIN, SULFITE EXPORTER TAU-E that are up- and down-regulated under mycorrhization, respectively; two genes HAEMOGLOBIN 2, SPERMIDINE HOC and C2H2 ZINC FINGER LIKE, MATE EFFLUX up- and down-regulated under nodulation, respectively. RT-qPCR data are the averages of three biological replicates (*n*>9). Statistical significance of differences between mycorrhized and nodulated roots was determined using an unpaired two-tailed Student’s *t*-test (***P<0.001). Error bars represent means ± SEM.

To validate the RNA-Seq results, 12 DEGs with different expression patterns were selected for RT-qPCR analysis (S1 Table) and then compared these results with the transcriptomic data obtained through RNA-Seq. AMF induces the expression of the GRAS-domain transcription factor RAM1 [17], and RAM1 induces RAM2, which produces cutin monomers during appressorium formation [18]. Infection and nodulation induces the expression of ERN1 (ETHYLENE-RESPONSIVE BINDING DOMAIN FACTOR REQUIRED FOR NODULATION) [58] and EARLY NODULATION 40 (ENOD40) [12]. We obtained similar results from both RT-qPCR and RNA-Seq analyses. RAM1 and RAM2 transcript levels were significantly increased in mycorrhized roots (Fig 4A), and ERN1 and ENOD40 transcripts were specifically induced in nodulated roots (Fig 4B). Further, ALC-INTERACTING 1 and AGAMOUS-LIKE 8 were found to be upregulated whereas; HEMOPEXIN and SULFITE EXPORTER TAU-E were downregulated under mycorrhization (Fig 4A). Similarly, HAEMOGLOBIN 2 and SPERMIDINE HOC were upregulated and C2H2 ZINC FINGER LIKE and MATE EFFLUX downregulated under nodulation conditions (Fig 4B). These results were consistent with those observed in RNA-Seq data of mycorrhized roots (Fig 4C) and nodulated roots (Fig 4D). Thus, the RNA-Seq results were considerably reliable for the identification of DEGs during mycorrhizal or rhizobial colonization in this study.

### Comparison of gene expression profiles in roots colonized by AMF and rhizobia

#### Defense-responsive genes

**Fig 5:**
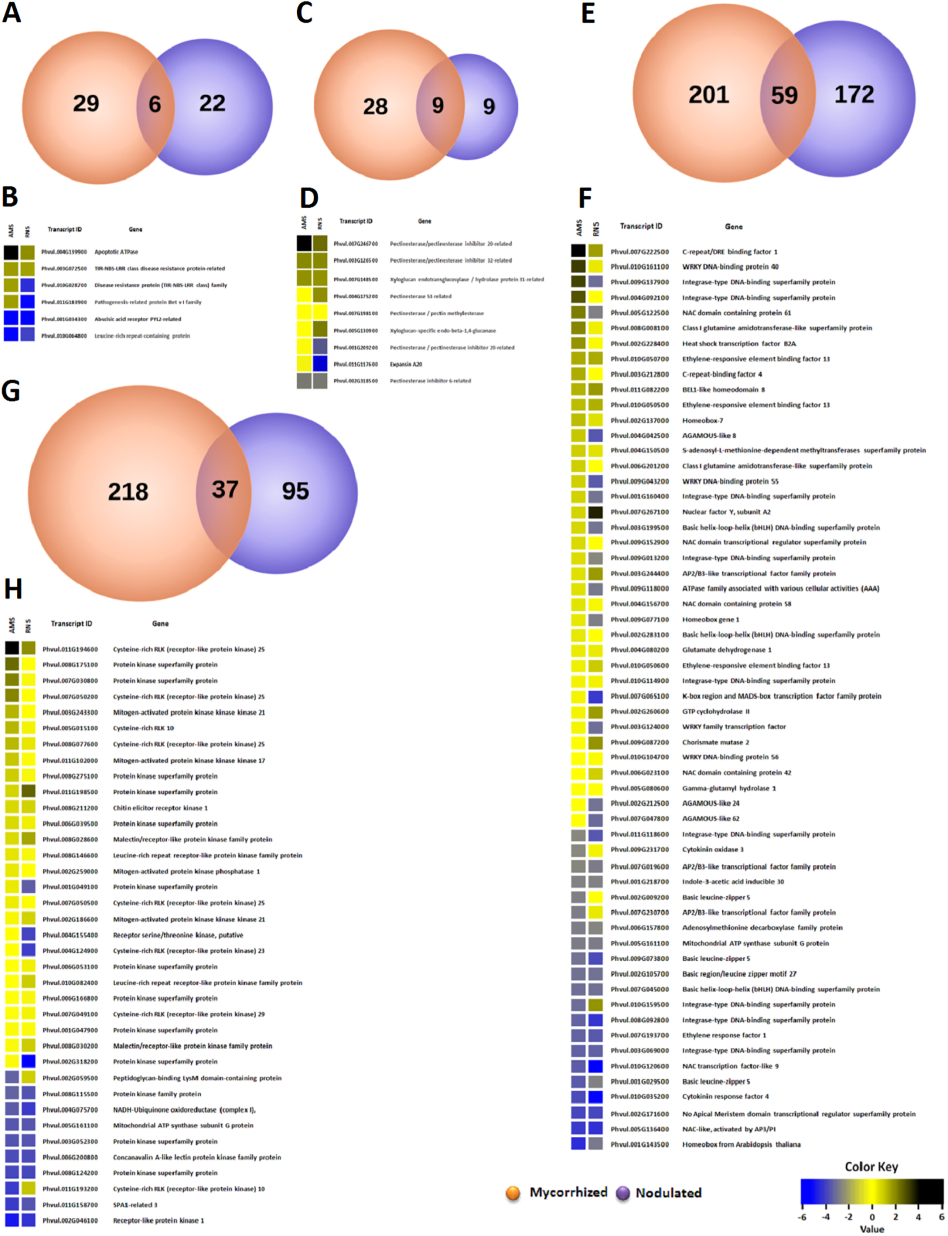
RNA-Seq based transcriptome comparison of genes induced by AMF and rhizobia in *P*. *vulgaris* roots. Differentially expressed genes upregulated and downregulated in response to AMF and rhizobia in *P*. *vulgaris* roots were identified based on *P*-values of ≤0.05 and fold-change of ≥2.0 (upregulated and downregulated). Unique and shared genes were identified after a pairwise comparison between treatments. Venn diagrams representing the number of DEGs involved in processes related to (A) defense, (C) cell wall, (E) nitrogen metabolism, and (G) phosphate metabolism. Heat maps of the overlapping gene expression patterns during mycorrhizal and rhizobial colonization for processes related to (B) defense, (D) cell wall, (F) nitrogen metabolism, and (H) phosphate metabolism. Color bar shows the fold-change range, with red and green representing downregulation and upregulation, respectively.

Plants mobilize strong defense responses to effectively ward off pathogens [59]. Plants also engage in symbiotic relationships with advantageous microorganisms such as root-associated bacteria or fungi, which enhance nutrient resources for plants [60,47]. These symbiotic relationships become established by the induction or suppression of mechanisms associated with plant defense. To elucidate changes in host plant defenses induced by AMF or nodule symbioses, we assessed DEGs in mycorrhized and nodulated roots using AgriGO and REVIGO. Based on GO analysis we observed a total of 35 and 28 differentially responding defense genes in mycorrhized and nodulated roots, respectively. The Venn diagram intersection revealed 51 unique (29 in AMF and 22 in rhizobial symbioses) and 6 overlapping defense genes in the colonized roots (Fig 5A). Among the unique genes, 28 were upregulated and 1 was downregulated in mycorrhized roots, whereas 3 were upregulated and 19 were downregulated in nodulated roots (S4A and S5 Figs and S6 Table). Interestingly, 23 unique genes of mycorrhized roots and 16 unique genes of nodulated roots belonging to disease resistance protein family (TIR-NBS-LRR class) were found upregulated and downregulated, respectively. Similarly, two unique genes of MLP-like protein family members were upregulated while mycorrhization and downregulated during nodulation (S5 Fig, S5 Table). Among the overlapping genes, one TIR-NSB-LRR family member (Phvul.010G028700) and one BET V I family member (Phvul.011G183900) were upregulated during mycorrhizal colonization and downregulated during rhizobial colonization (Fig 5B). The observation that these two genes have opposite expression patterns during the two symbioses suggests that these genes could act as defense markers during symbiotic interactions. We also analyzed the known genes associated with *P*. *vulgaris* defense from the jasmonic acid (JA) and salicylic acid (SA) KEGG signaling pathways (plant signal transduction). The JASMONATE ZIM-DOMAIN (JAZ) genes are important repressors of the JA signaling pathway [61]; they were upregulated during mycorrhization and were variably expressed (i.e., exhibited both upregulation and downregulation of different transcripts of the same gene) during nodulation. The expression of JASMONATE RESISTANCE (JAR1), a pathogen defense gene [62], was downregulated during mycorrhizal colonization and upregulated during nodulation (S6 Fig; S6 Table). NPR1 was variably expressed in AMF-colonized roots and upregulated in nodulated roots compared with controls. Downstream genes such as TGA and PR-1 were variably expressed during both types of symbioses, except PR-1 was suppressed during mycorrhizal colonization compared with the control (S6 Fig; S6 Table). These results suggest that different isoforms of defense genes are elicited in *P*. *vulgaris* roots during AMF and rhizobial symbioses.

#### Cell wall-related genes

The plant cell wall acts as a physical barrier against invading pathogens and parasites [63]. By contrast, the cell wall is modified during penetration and establishment of the symbiotic interface in AMF and rhizobial symbioses [64]. In the present study, based on GO analysis we observed 37 and 18 cell wall-related genes that were differentially regulated (upregulation and downregulation fold-change of ≥2.0) in *P*. *vulgaris* roots during AMF and rhizobial symbioses, respectively. A total of 21 genes were upregulated and 15 were downregulated in AMF-colonized roots, whereas 12 were upregulated and 5 were downregulated during nodulation (S4B and S7 Figs). The Venn diagram intersection identified 9 overlapping genes, 28 unique genes during mycorrhizal colonization, and 9 unique genes during nodulation (Fig 5C). These belong to different enzyme isoforms that target the plant cell wall by wall-loosening activities, including PECTIN-RELATED genes, PLANT INVERTASE and XYLOGLUCAN ENDOTRANSGLUCOSYLASE/HYDROLASE (XTH). On the other hand, expansin proteins non-enzymatically trigger a pH dependent relaxation of the cell wall which loosens and softens during cell expansion. Among the overlapping genes, XYLOGLUCAN-SPECIFIC ENDO-BETA-1,4-GLUCANASE (Phvul.005G130900) and PECTINESTERASE 53-RELATED (Phvul.004G175200) genes were downregulated during mycorrhizal colonization and upregulated during nodulation. We observed that EXPANSIN A20 was downregulated under both symbiotic conditions (Fig 5D, S6 Table). Among the unique genes, EXPANSIN-LIKE A2 and EXPANSIN B3 were upregulated, whereas EXPANSIN A7 and EXPANSIN A18 were downregulated under mycorrhization; EXPANSIN A15, EXPANSIN-LIKE B1 and B2 were upregulated under nodulation (S7 Fig; S6 Table). We observed that EXPANSIN A20 was downregulated and EXPANSIN-LIKE B2 was upregulated under both symbiotic conditions (Fig 5D, S7 Fig). Ten and nine unique genes belonging to PLANT INVERTASE/PECTIN METHYLESTERASE INHIBITOR superfamily and XTH family are the most abundant class of enzymes found in mycorrhized roots, respectively. Among the unique genes of nodulation, only two members for each of these enzymes were differentially expressed (S7 Fig; S6 Table). Pectin methylesterase is post-transcriptionally regulated by endogenous protein inhibitors and functions to maintain cell wall integrity in plant immunity whereas, xyloglucan endotransglucosylase enzyme cleaves and religates xyloglucan polymers and is an essential constituent of the primary cell wall in growing tissues [65,66]. These combined results indicate that several enzyme isoforms regulating cell wall remodeling in *P*. *vulgaris* are uniquely expressed during these symbioses, thereby modifying cell wall plasticity during plant–symbiont interactions.

#### Nitrogen compound metabolism

Nitrogen metabolism is an important pathway in plants, and it is specifically crucial for nodulating plants. We compared the expression of genes involved in N metabolism in *P*. *vulgaris* roots during AMF and rhizobial symbioses, and identified a total of 260 DEGs in AMF symbiosis and 231 DEGs in rhizobial symbiosis based on GO analysis. Of these, 173 genes were upregulated and 87 were downregulated in mycorrhization, whereas 112 genes were upregulated and 119 were downregulated during nodulation (S4C and S8 Figs). We identified 59 overlapping genes involved in N metabolism in the two symbiotic interactions (Fig 5E); of these, 38 and 29 genes were upregulated and 21 and 30 were downregulated during AMF and rhizobial symbioses, respectively (Fig 5F).

Recent work demonstrated that the AP2 transcription factor is a key player in soybean nodule initiation [67]. Nodule initiation and differentiation are controlled by the ETHYLENE-RESPONSIVE TRANSCRIPTION FACTOR 1 (ERF1 [68]) in *L*. *japonicus* and the EDF [69] and MADS-BOX transcription factors NGL9 and NMH7 [70] in *Medicago* species. A recent study by Azarakhsh et al. [71] reported that a homeobox transcription factor (KNOTTED1-LIKE) regulates cytokinin biosynthesis during nodule development in *M*. *truncatula*. However, little is known about the role of these genes during AMF symbiosis. In this study, we found that most of the N metabolism genes belong to different transcription factor families (Fig 5F, S8 Fig). Unique genes and different homologs of the AP2 DOMAIN, ERF, MADS-BOX genes, and HOMEOBOX transcription factors (S5 Table) were differentially expressed in *P*. *vulgaris* roots during AMF and rhizobial symbioses (S8 Fig). Among the overlapping genes, the AP2/B3 TFs such as Phvul.003G244400 is upregulated, Phvul.007G019600 is downregulated in both symbioses. Whereas Phvul.007G230700 (AP2/B3 TF) is downregulated during mychorrhiza colonization and Phvul.007G065100 (MADS-BOX) is downregulated during rhizobia colonization (Fig 5F; S6 Table), indicating that gene expression in the host root is differentially elicited depending on the type of symbiosis. During nitrogen fixation in tropical legumes like bean, the ammonia is reduced in uninfected cells via purine oxidation to the ureides allantoin and allantoate [72]. These ureides are the main forms of organic nitrogen transported and stored in plants [73]. As anticipated, genes involved in purine biosynthesis (PHOSPHORIBOSYL PYROPHOSPHATE AMIDOTRANSFERASE, GLYCINE LIGASE, and PHOSPHORIBOSYL AMINOIMIDAZOLE CARBOXYLASE), ureide biosynthesis (URICASE, XANTHINE DEHYDROGENASE 1, and ALLANTOINASE), and ammonia assimilation [GLUTAMINE SYNTHETASE and NADH-DEPENDENT GLUTAMATE SYNTHASE 2 (GOGAT)] were upregulated only in nodulated roots (S3 Table and S8 Fig). We also analyzed the gene expression levels of all known key enzymes (15 genes) in the *P*. *vulgaris* KEGG nitrogen metabolism pathway. During mycorrhizal symbiosis, 8 of 15 gene transcripts were upregulated, 4 were downregulated, and 3 exhibited variable expression (S9A Fig; S6 Table). During rhizobial symbiosis, 12 of 15 genes were upregulated and 3 exhibited variable expression (S9B Fig; S6 Table). These gene profiles reflect different transcript abundance levels during the *P*. *vulgaris* response to AMF and rhizobia. The key genes and enzymes identified in the KEGG nitrogen metabolism pathway are strongly induced in response to rhizobial colonization, which primarily involves biological nitrogen fixation.

#### Phosphorus metabolism

The macronutrient P is crucial for the biosynthesis of key cellular biomolecules, and plants cannot grow without a reliable P supply. Inorganic phosphate (P_i_) is involved in controlling key enzyme reactions and regulating metabolic pathways [74]. AMF aid the host plant with P_i_ uptake. Therefore, we compared the expression of genes involved in P metabolism during mycorrhizal and rhizobial colonization. Based on GO analysis we observed a total of 255 and 132 DEGs involved in P metabolism in response to mycorrhizal and rhizobial colonization, respectively (Fig 5G). Of these, 203 genes were upregulated and 52 were downregulated during mycorrhizal colonization, whereas 62 genes were upregulated and 70 were downregulated during rhizobial colonization (S4D Fig). The Venn diagram intersection identified 37 overlapping genes (Fig 5H) and 218 and 95 unique genes, respectively, during mycorrhizal and rhizobial colonization (S10 Fig). Among the overlapping genes, WALL-ASSOCIATED RECEPTOR KINASE C-TERMINAL (Phvul.001G049100, Phvul.002G318200), SALT STRESS RESPONSE/ANTIFUNGAL (Phvul.004G124900), and GLYCEROPHOSPHODIESTER PHOSPHODIESTERASE (GDPD; Phvul.004G155400) were upregulated during mycorrhizal symbiosis and downregulated during rhizobial symbiosis (Fig 5H). Conversely, a NON-SPECIFIC SERINE/THREONINE PROTEIN KINASE (Phvul.002G059500) was upregulated during rhizobial symbiosis and downregulated during mycorrhizal symbiosis. Isoforms of LECTIN-LIKE genes were found to be the most abundant class of upregulated unique genes in mycorrhized roots (S10 Fig). Several studies reported that lectin genes are expressed in arbuscule-containing cells in *M*. *truncatula* [75–77].

Next, we assessed the expression of 23 known genes and enzymes identified in the *P*. *vulgaris* KEGG inositol phosphate (IP) metabolism pathway; these are involved in regulating many cellular functions such as cell growth, apoptosis, endocytosis, and cell differentiation [78]. The transcripts of INOSITOL-PENTAKISPHOSPHATE 2-KINASE (IPK1) (involved in phytate biosynthesis, a phosphorous reservoir [79]), 1D-MYO-INOSITOL-TRIPHOSPHATE 3-KINASE, INOSITOL-1,4,5-TRISPHOSPHATE 5-PHOSPHATASE, and MYOTUBULARIN ISOFORMS [80] were all upregulated in mycorrhized roots (S11A Fig; S6 Table) and downregulated during nodulation (S11B Fig; S6 Table). The transcripts of INOSITOL-POLYPHOSPHATE MULTIKINASE family genes were downregulated in mycorrhized roots and upregulated during nodulation. These results confirm that most genes involved in P and IP metabolism are upregulated in *P*. *vulgaris* roots in response to mycorrhizal symbiosis, conditions that promote P uptake, compared with rhizobial symbiosis.

## Discussion

*Phaseolus vulgaris* is one of the most extensively studied model legumes in the world. There has been a focus on gene functional characterization in recent years, specifically in the area of *Phaseolus-*rhizobia symbiosis [21,26–30]. However, studies on *Phaseolus-*AMF symbiosis are limited [33,81]. The availability of genetic resources such as expressed sequence tags [82] and global gene expression profiles [83] stimulated rapid advances in rhizobial symbiosis research. There are fewer resources for AMF symbiosis research in *Phaseolus*, and there is an urgent need for their development. Some studies in other legumes have investigated symbiosis-induced responses in roots colonized by AMF and rhizobia using transcriptome data obtained from cDNA macroarrays and microarrays. Those studies selected samples for comparative transcriptomic analysis based on symbiotic structures in the roots of *L*. *japonicus* (27 dpi with AMF and 12 dpi with rhizobia [84]), *M*. *truncatula*, and *C*. *gluca* (45 dpi with AMF [37]). In the present study, systematic analyses were performed to identify an ideal symbiotic stage for investigating both mycorrhizal and rhizobial associations with wild-type *P*. *vulgaris* roots. At 2 wpi, mycorrhizal and rhizobial symbionts established mature symbiotic structures and were physiologically active in P uptake and N fixation, respectively. An RNA-Seq-based gene expression analysis in control, mycorrhized, and nodulated roots of *P*. *vulgaris* using Ion Proton sequencing provided a global gene expression profile under each condition. Statistics and observed expression levels were used to identify the DEGs (upregulated and downregulated) for mycorrhized and nodulated roots; subsequently, the data sets were compared. These combined analyses identified 3,219 DEGs (fold-change cut-off of ≥2.0 upregulated and downregulated) in response to mycorrhizal symbiosis and 2,645 in response to rhizobial symbiosis. RNA-Seq analyses identified a total of 3,641 DEGs during AMF development (27 dpi [84]) in *L*. *japonicus*, and 1,668 DEGs during nodulation (21 dpi) in *P*. *vulgaris* [83]. The RNA-Seq approach identified more DEGs than previous microarray studies in AMF- or arbuscule-containing cells [85–89], possibly because RNA-Seq provides more precise measurements of transcript levels and their isoforms, and it has a broader dynamic range than microarray methods [40]. The gene expression data obtained by our RNA-Seq analysis were validated for 12 DEGs with different expression patterns in *P*. *vulgaris* using RT-qPCR. Quantitative RT-PCR results were consistent with those observed in RNA-Seq data of mycorrhized and nodulated roots. For instance, the relative expression levels of the AMF-specific GRAS-domain transcription factor RAM1, RAM1-induced RAM2, and rhizobial-specific ERN1 and ENOD40 displayed similar fold-change expression trends using RNA-Seq. The same expression trends of RAM1, ERN1, and ENDO40 in *P*. *vulgaris* [32,34] and RAM2 in *M*. *truncatula* [18] were previously reported as symbiotic marker genes in legumes. The AgriGO platform was used to derive a total of 180 and 160 significant GO categories for mycorrhizal and rhizobial symbioses, respectively, including biological process, molecular function, and cellular component. Protein metabolism, other metabolic processes, transport, signal transduction, transcription factor activity, kinase activity, defense, cell wall and plasma membrane were few among the most abundant GO terms found in mycorrhizal and rhizobial symbioses samples. High similarity was identified previously in GO term enrichment data by Handa et al. [84] in mycorrhized roots of *L*. *japonicus* and *M*. *truncatula* roots treated with nod factor [90]. Our primary focus was to identify unique and shared genes involved in defense, cell wall-related processes, N metabolism, and P metabolism in mycorrhizal and rhizobial symbiosis.

To establish symbiotic interactions, host plants repress gene families involved in defense reactions. A previous transcriptome analysis using RNA-Seq in rhizobial-inoculated *M*. *truncatula* roots treated with the nitric oxide synthase inhibitor cPTIO [2-(4-CARBOXYPHENYL)-4,4,5,5-TETRAMETHYLIMIDAZOLINE-1-OXYL-3-OXIDE] detected downregulation of defense genes elicited by rhizobial inoculation [91]. Similarly, the expression profiles of arbuscule-containing cells indicated that some DEFENSIN and CHITINASE genes [87] are elicited, and some defense responses are suppressed [89,92]. Specific TIR-NBS-LRR proteins negatively regulate the jasmonic acid signalling genes [93], and such low JA signalling encourage AM fungal colonization in plants [94]. Herein, we found that TIR-NBS-LRR family genes were most abundant class of defence genes that differentially expressed in both symbioses. Interestingly, upregulation of 23 TIR-NBS-LRR members unique to mycorrhization could result in decreased expression of JAR1 which may be involved in suppression of defence against invading AM fungi. Whereas, rhizobial colonization downregulated all the 16 unique TIR-NBS-LRR members therefore JAR1 expression was found upregulated which perhaps evoked the defence that might recognize the rhizobia as symbiont for successful colonization. Nevertheless, this hypothesis needs to be demonstrated. *Arabidopsis* JAR1 was upregulated in response to infection with the fungal pathogen *Botrytis cinerea* [95] and the bacterial pathogens *Pseudomonas syringae* and *Ralstonia solanacearum*, which activated the JA signaling pathway as a defense response [94]. In contrast to the response to the pathogenic fungus *B*. *cinerea*, we observed here that JAR1 expression was suppressed during mycorrhizal symbiosis. Although JAR1 is upregulated in nodulated roots, some PR-1 genes (salicylic acid-mediated defense) were variably expressed, indicating that rhizobial surface polysaccharides could suppress or help to evade this first basal defense [96,97]. Our combined results suggest that different defense gene isoforms (both unique and shared genes) exhibited differential responses to mycorrhizal and rhizobial symbioses.

Cell wall remodeling during root symbioses with microsymbionts facilitates the establishment of a close interface that enables developmental coordination and nutrient exchange. Cell wall remodeling accommodates plant cell growth, which begins with selective cell wall loosening, followed by water uptake and cell enlargement [98]. During mycorrhization and nodulation, several isoforms of cell wall-loosening genes such as PECTINS and XYLOGLUCAN ENDOTRANSGLUCOSYLASE/HYDROLASES were differentially expressed. Rich et al. [64] reported that symbiotic signals induced cell wall modifications during penetration and establishment of the symbiotic interface during nodulation and mycorrhization. In *Medicago* XYLOGLUCAN ENDOTRANSGLUCOSYLASE/HYDROLASES 1 (XTH1) promoter was highly active in mycorrhizal roots and its transcripts were significantly induced in upon mycorrhization [99]. Similarly, herein we observed XTH family proteins are one of the most abundantly expressing genes unique to mycorrhizal colonization suggesting its specificity to fungal symbiosis. A study in soybean shows that the root hair specific EXPANSIN B1 regulates formation of root hairs whereas; EXPANSIN B2 controls the root hair elongation [100]. Based on the fact that rhizobia invade the root via root hair cells [12] and interestingly in our study the unique genes of nodulated roots shows both EXPANSIN B1 and B2 transcripts were upregualted indicating its possible involvement in rhizbobial invasion into the host root. This notion was supported by a separate study where β-EXPANSIN gene expression profiles have been characterized during the early stage of mycorrhization and nodulation in *L*. *japonicus*, *Glycine max* (soybean), and *Melilotus officinalis* (sweet clover) [101]. Our study showed that unique EXPANSIN genes in *P*. *vulgaris* responded differentially to mycorrhizal and rhizobial symbioses. We also found that several unique cell wall-related genes involved in softening and remodeling the *P*. *vulgaris* root were differentially expressed during mycorrhizal and rhizobial symbioses.

Transcriptomic approaches provide powerful tools to investigate complex network interactions in N metabolism, which involves N uptake and regulation, amino acid metabolism, and N translocation and remobilization [102–104]. Several N metabolism genes that participate in rhizobial symbiosis have been characterized, such as MsNGL9 and MsNMH7 [70], LjERF1 [68], MtEDF [69], GmAP2 [67], and MtKNOTTED1-LIKE [71]. Genes encoding NF-Y subunits act as components of a hierarchical transcriptional activation cascade in the nodulation signaling pathway [21,25,104–106]. Reduction of PvNF-YC1 [25] and LjNF-YB [21] transcript levels by RNA interference leads to the arrest of nodule development and defective rhizobial infection. Genes involved in purine and ureide biosynthesis and ammonia assimilation have key roles in transport and storage of organic nitrogen in host plants [73]. Results from our present analysis support these observations, as bean homologs of these genes were upregulated during rhizobial symbiosis. We also obtained the expression profiles of N metabolism genes during mycorrhizal symbiosis. We observed that most genes involved in N metabolism in mycorrhized roots belong to different transcription factor families; however, functional characterization studies are required to decipher their roles during AMF symbiosis.

In plants, depletion of P_i_ induces dramatic changes in developmental and metabolic programs and in transcriptomic, proteomic, and metabolomic profiles. AMF symbiosis can reduce the effects of P_i_ depletion in the rhizosphere, thereby improving plant P nutrition and growth [107]. The evolutionarily conserved P metabolism gene GDPD has an important role in several physiological processes in both prokaryotes and eukaryotes. *Arabidopsis* GDPD maintains cellular phosphate homeostasis under phosphate starvation [108]. We observed that GDPD was upregulated in mycorrhized *P*. *vulgaris* roots. AM-specific lectins are known to be incorporated into plant cell walls [77], and several isoforms of lectin-like proteins were induced in AMF-colonized roots. Some genes for lectin-like proteins were also upregulated during rhizobial symbiosis, although their functions have not been determined. Among the known genes and enzymes involved in the *P*. *vulgaris* inositol phosphate metabolism pathway, we observed that IPK1, 1D-MYO-INOSITOL-TRIPHOSPHATE 3-KINASE, INOSITOL-1,4,5-TRISPHOSPHATE 5-PHOSPHATASE, and different MYOTUBULARIN ISOFORMS were upregulated in AMF-colonized roots. IPK1 is implicated in the synthesis of phytate, a phosphate storage form that is used to maintain phosphate homeostasis in *Arabidopsis* [79]. Our combined data confirm that most genes involved in P and IP metabolism are upregulated in mycorrhized roots, which are primed to promote P uptake [74].

## Conclusions

We performed comparative transcriptome analyses of *P*. *vulgaris* roots colonized by AMF and rhizobia, and identified differentially expressed unique and shared genes associated with defense, cell wall structure, N metabolism, and P metabolism. The identified DEGs may be involved in the formation of these beneficial symbiotic relationships. However, further research is required to determine the putative roles of these DEGs during specific symbiotic interactions. These investigations will extend our understanding of symbiotic genetic signaling networks, and identify candidate genes that could be targeted to enhance P uptake and N-fixing capacity, and thereby increase the net yield of this valuable grain legume.

## Supporting information

S1 Fig Overview of experimental design for RNA sequencing and data analysis. (PDF)

S2 Fig Heatmap showing hierarchical clustering based on expression values of transcripts in control, mycorrhized, and nodulated root samples. (PDF)

S3 Fig Interactive graph of biological processes and molecular function GO terms, as determined by AgriGO and REVIGO algorithms. (PDF)

S4 Fig Graphical representation of number of unique upregulated and downregulated genes that responded during mycorrhizal and rhizobial colonization. (PDF)

S5 Fig DEGs of defense-responsive genes during root symbioses. (PDF)

S6 Fig Effect of root symbiosis on plant signal transduction pathways in mycorrhized and nodulated *P*. *vulgaris* roots. (PDF)

S7 Fig DEGs of cell wall-related genes during root symbioses. (PDF)

S8 Fig DEGs of N metabolism genes during root symbioses. (PDF)

S9 Fig Effect of root symbiosis on the nitrogen metabolism pathway in mycorrhized and nodulated roots. (PDF)

S10 Fig DEGs of P metabolism genes during root symbioses. (PDF)

S11 Fig Effect of root symbiosis on the inositol phosphate metabolism pathway in mycorrhized and nodulated *P*. *vulgaris* roots. (PDF)

S1 Table Primer sequences of *P*. *vulgaris* genes used to perform RT-qPCR analyses. (DOCX)

S2 Table Summary of the number of Ion Proton sequencer reads and BLASTX hits to the *P*. *vulgaris* genome database. (DOC)

S3 Table List of upregulated and downregulated DEGs identified from mycorrhized and nodulated *P*. *vulgaris* roots. (XLS)

S4 Table List significantly enriched GO terms of upregulated and downregulated DEGs identified from mycorrhized and nodulated *P*. *vulgaris* roots. (XLS)

S5 Table Total number of transcription factors of different families that are differentially expressed in mycorrhized and nodulated *P*. *vulgaris* roots. (DOCX)

S6 Table Quantitative expression profiles of unique and overlapping DEGs related to defense, cell wall, N metabolism and P metabolism. (XLS)
