# Supplementary material for: Differentially expressed genes in mycorrhized and nodulated roots of common bean are associated with defense, cell wall architecture, N metabolism, and P metabolism
Source: PLoS One. 2017 Aug 3;12(8):e0182328. doi: 10.1371/journal.pone.0182328 (PMC5542541; doi:10.1371/journal.pone.0182328)
Supplement: S1 Fig — (PDF) [file pone.0182328.s001.pdf]

S1 Fig

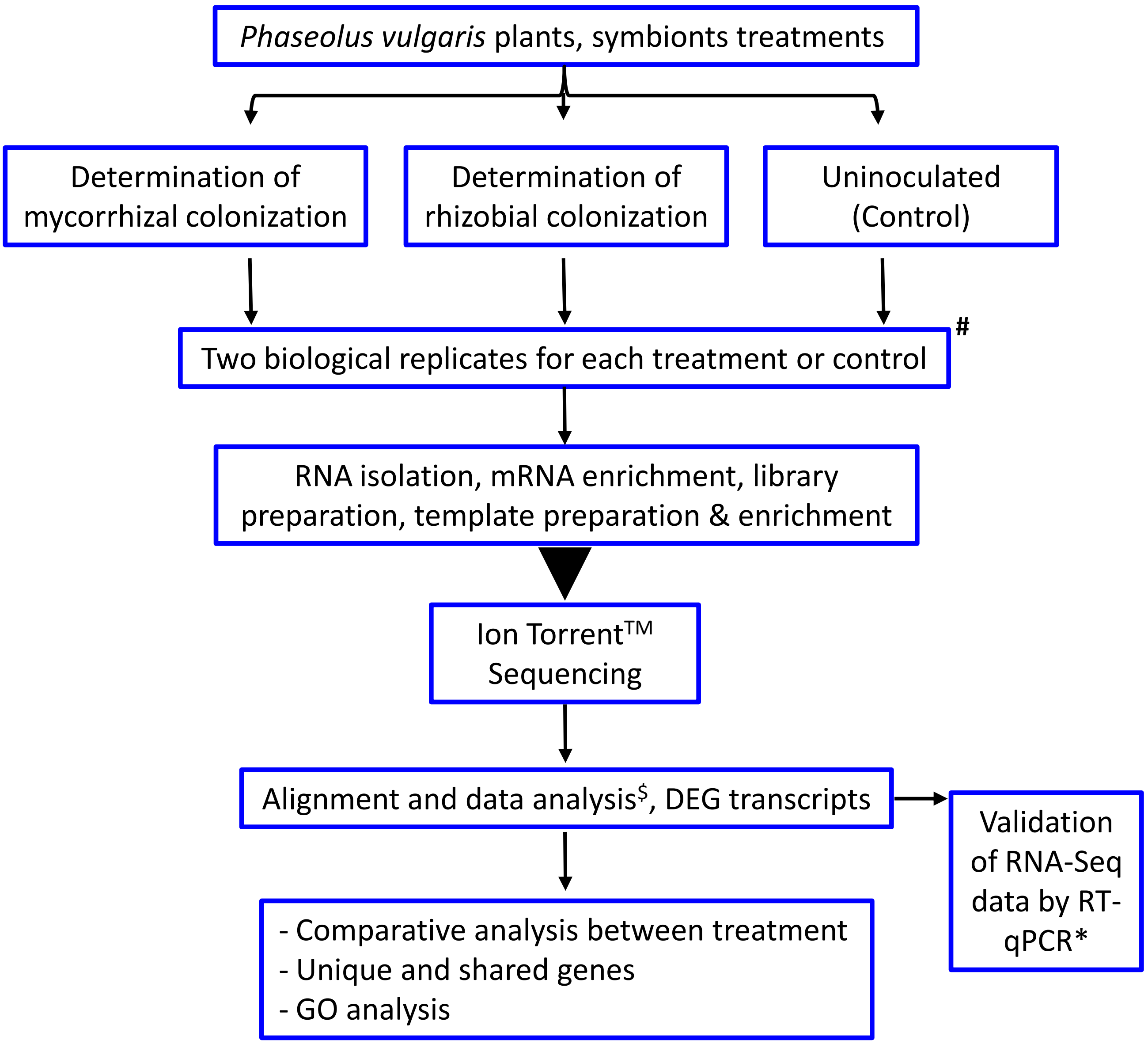

S1 Fig. Overview of experimental design for RNA-sequencing and data analysis. Five-day-old wild-type *P. vulgaris* plants were inoculated with *Rhizophagus irregularis* for mycorrhizal colonization or *Rhizobium tropici* for the establishment of root nodules. At 2 weeks post inoculation (wpi), the symbiont-colonized roots were subjected to global RNA sequencing analysis. # Each biological replicate was a pool of 3 symbiont-colonized or control roots. $ Data analysis includes normalization and quantification (fold-change in expression over control), and values were averaged for two replicates; * validation of RNA-Seq data by RT-qPCR form three biological replicates.
