# Supplementary material for: Differentially expressed genes in mycorrhized and nodulated roots of common bean are associated with defense, cell wall architecture, N metabolism, and P metabolism
Source: PLoS One. 2017 Aug 3;12(8):e0182328. doi: 10.1371/journal.pone.0182328 (PMC5542541; doi:10.1371/journal.pone.0182328)
Supplement: S2 Fig — (PDF) [file pone.0182328.s002.pdf]

S2 Fig

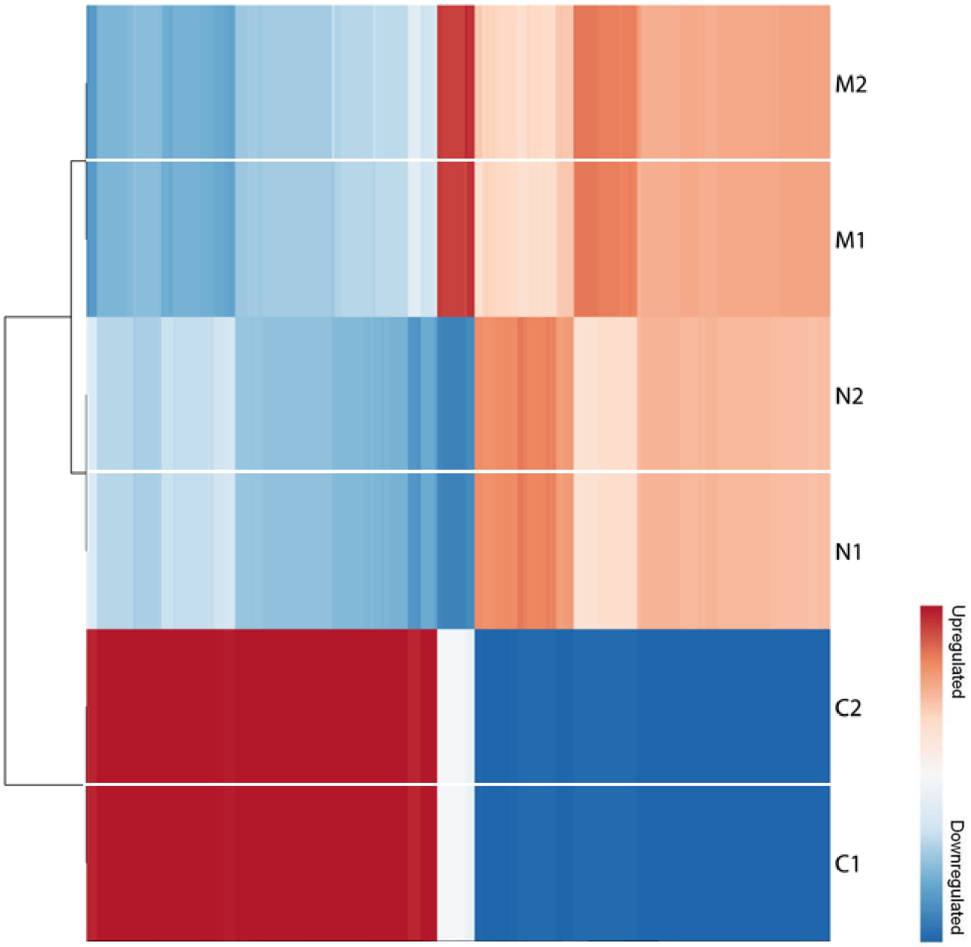

S2 Fig. Heatmap showing hierarchical clustering based on expression values of the transcripts in control, mycorrhized, and nodulated root samples. Expression data from two biological replicates (1 and 2) of mycorrhized (M), nodulated (N), and control (C) roots were used. Hierarchical clustering results were visualized using TreeView by Clustvis web tool.
