# Supplementary material for: Differentially expressed genes in mycorrhized and nodulated roots of common bean are associated with defense, cell wall architecture, N metabolism, and P metabolism
Source: PLoS One. 2017 Aug 3;12(8):e0182328. doi: 10.1371/journal.pone.0182328 (PMC5542541; doi:10.1371/journal.pone.0182328)
Supplement: S3 Fig — (PDF) [file pone.0182328.s003.pdf]

S3 Fig

Mycorrhized

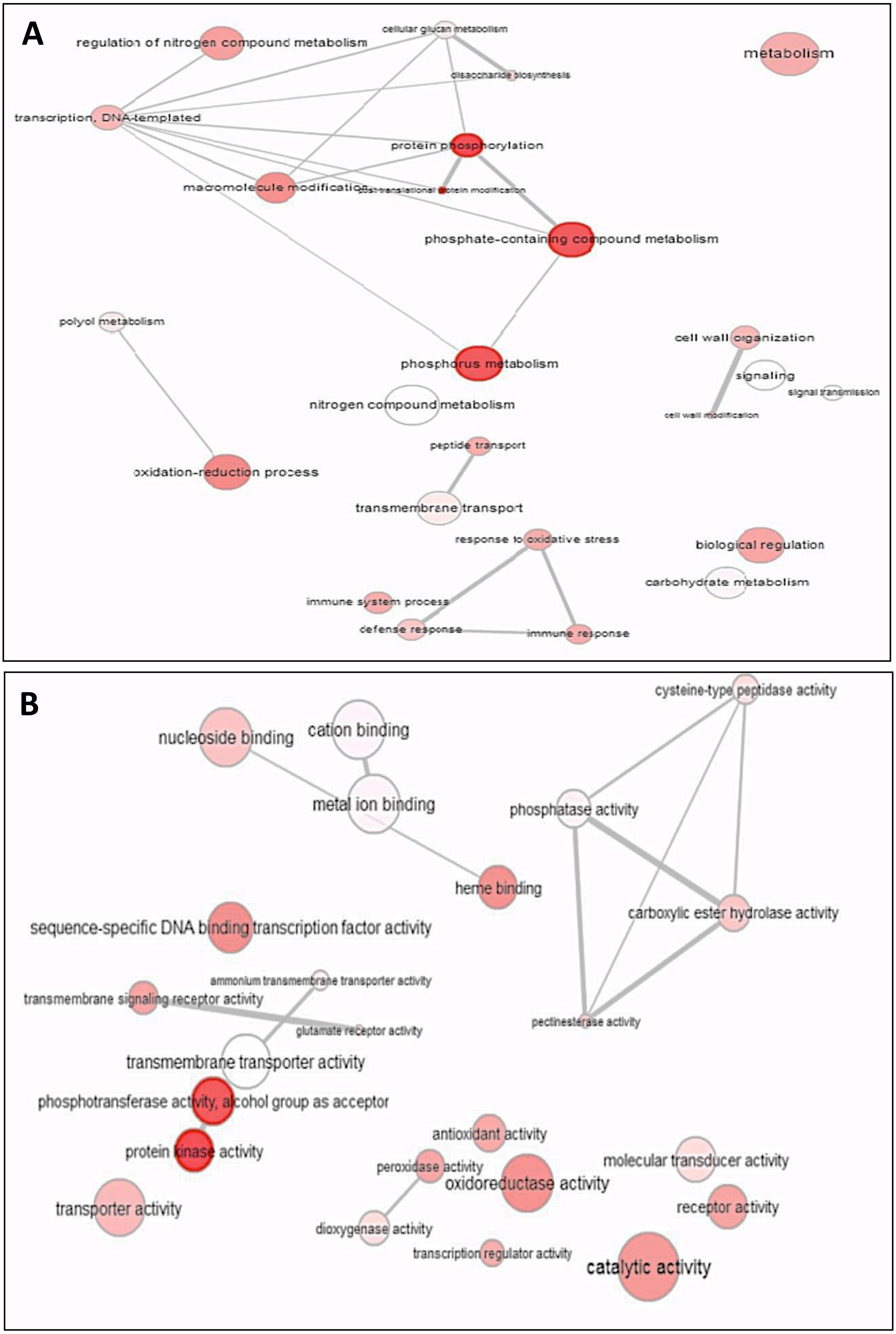

Nodulated

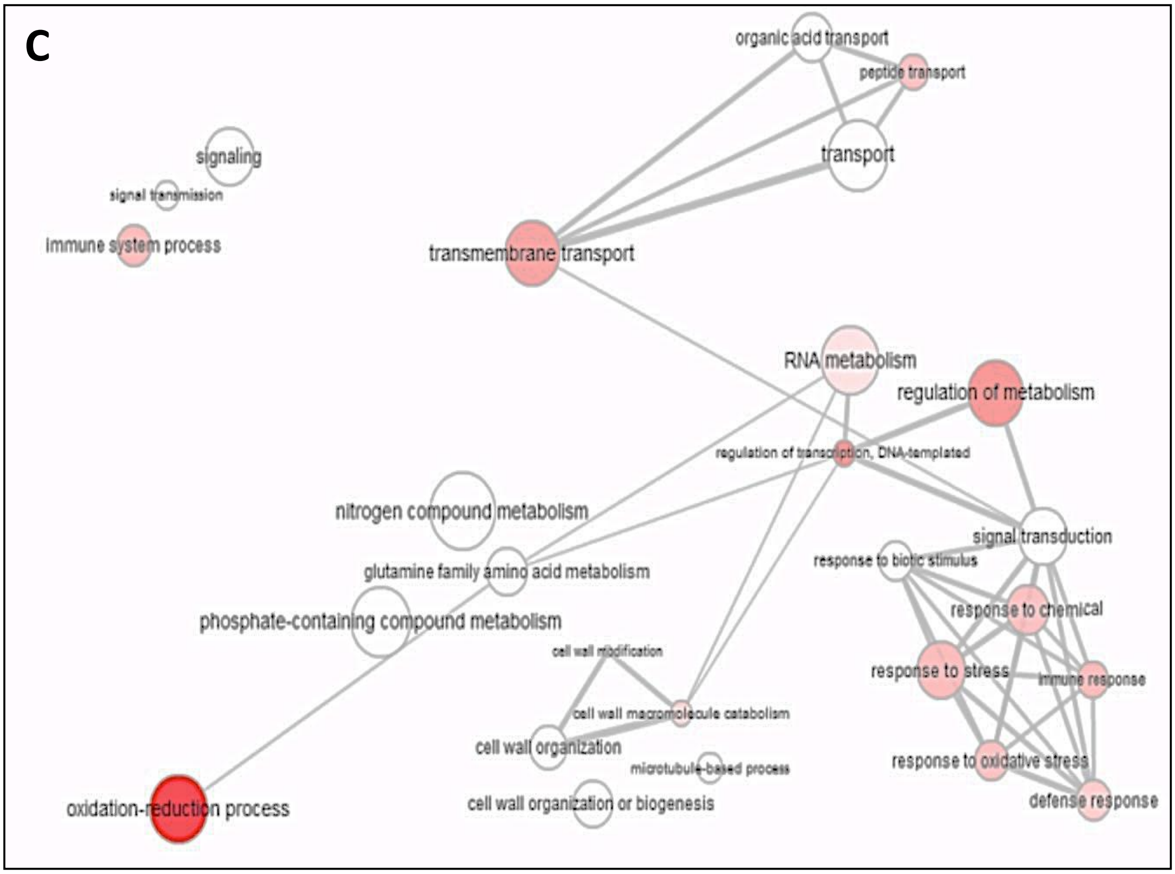
C
organic acid transport
peptide transport
transport
signaling
signal transmission
immune system process
transmembrane transport
RNA metabolism
regulation of metabolism
regulation of transcription, DNA-templated
nitrogen compound metabolism
signal transduction
response to biotic stimulus
glutamine family amino acid metabolism
phosphate-containing compound metabolism
response to chemical
cell wall modification
response to stress
immune response
cell wall macromolecule catabolism
cell wall organization
microtubule-based process
response to oxidative stress
oxidation-reduction process
cell wall organization or biogenesis
defense response

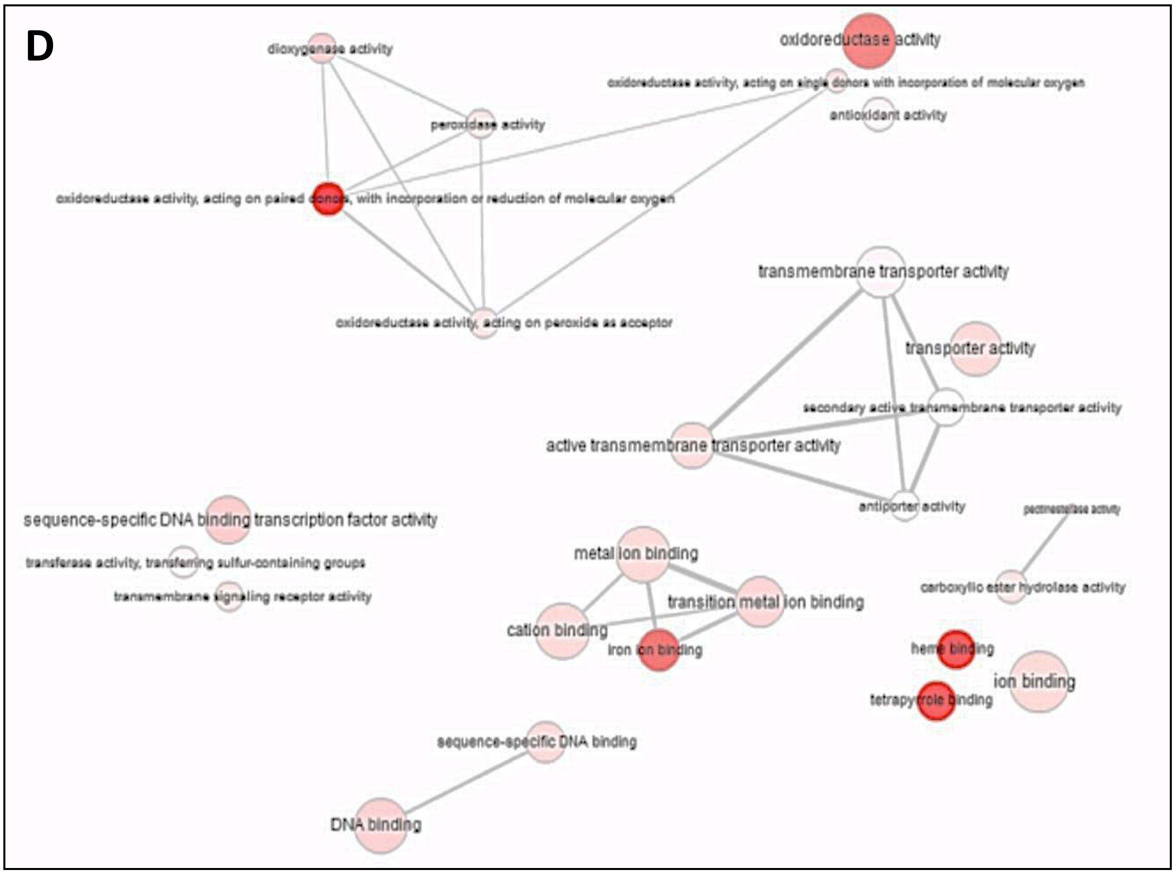
D
dioxygenase activity
oxidoreductase activity
oxidoreductase activity, acting on single donors with incorporation of molecular oxygen
antioxidant activity
peroxidase activity
oxidoreductase activity, acting on paired donors, with incorporation or reduction of molecular oxygen
transmembrane transporter activity
oxidoreductase activity, acting on peroxide as acceptor
transporter activity
secondary active transmembrane transporter activity
active transmembrane transporter activity
antiporter activity
sequence-specific DNA binding transcription factor activity
metal ion binding
carboxylic ester hydrolase activity
transferase activity, transferring sulfur-containing groups
transmembrane signaling receptor activity
transition metal ion binding
cation binding
iron ion binding
heme binding
ion binding
tetrapyrrole binding
sequence-specific DNA binding
DNA binding

S3 Fig. Interactive graph of biological processes and molecular function GO terms, as determined by AgriGO and REVIGO algorithms. GO terms are represented by bubbles and are plotted according to semantic similarities to other GO terms (bubbles linked by edges are most closely related). Bubble size indicates the frequency of the GO term; color indicates semantic similarity. In mycorrhized roots, GO category (A) biological process and (B) molecular function. In nodulated roots, GO category (C) biological process and (D) molecular function.
