# Supplementary material for: Differentially expressed genes in mycorrhized and nodulated roots of common bean are associated with defense, cell wall architecture, N metabolism, and P metabolism
Source: PLoS One. 2017 Aug 3;12(8):e0182328. doi: 10.1371/journal.pone.0182328 (PMC5542541; doi:10.1371/journal.pone.0182328)
Supplement: S4 Fig — (PDF) [file pone.0182328.s004.pdf]

S4 Fig

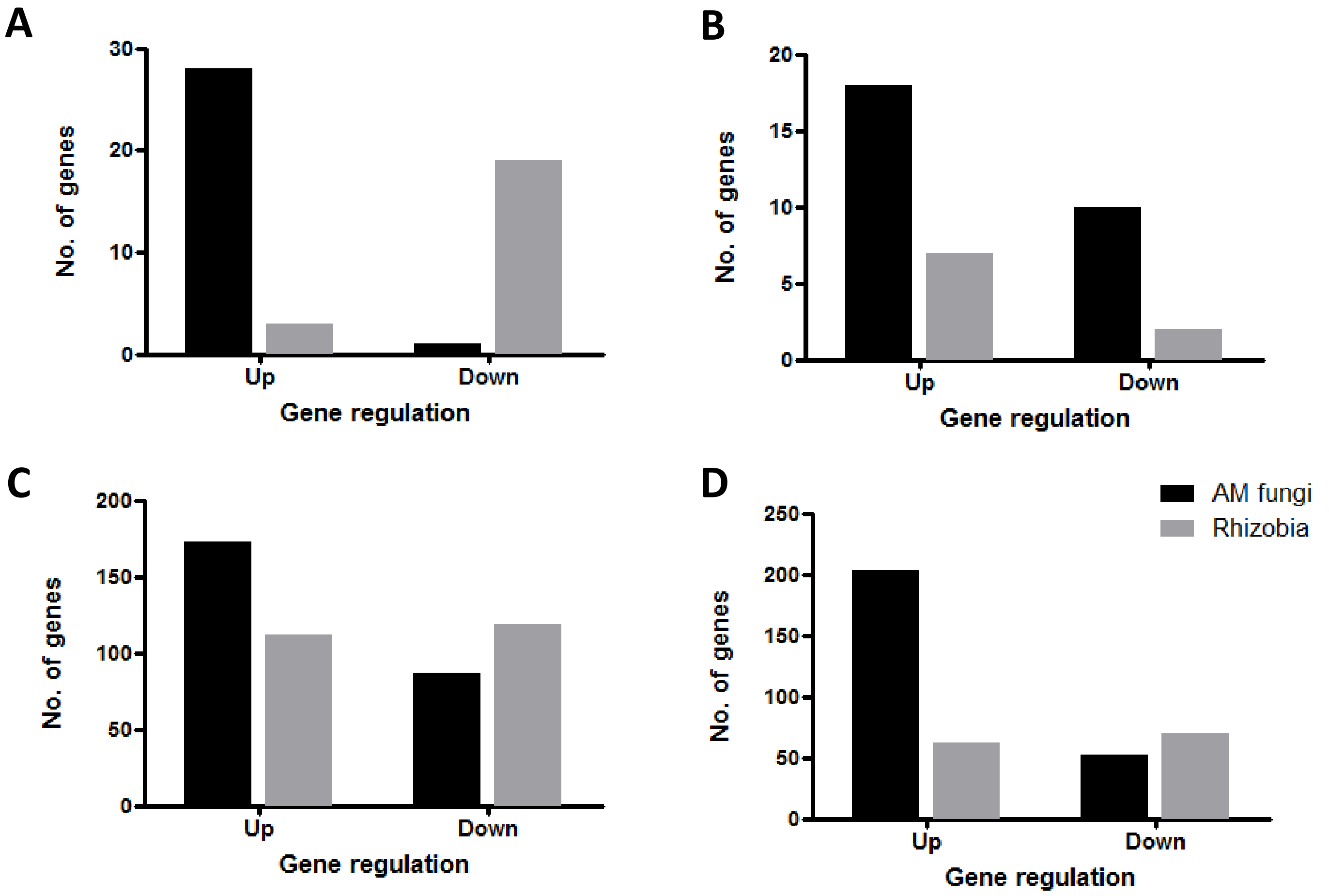

S4 Fig. Graphical representation of the number of unique upregulated and downregulated genes that responded during mycorrhizal and rhizobial colonization. (A) Defense-responsive, (B) cell wall-related, (C) nitrogen metabolism, and (D) phosphate metabolism genes.
