# Supplementary material for: Differentially expressed genes in mycorrhized and nodulated roots of common bean are associated with defense, cell wall architecture, N metabolism, and P metabolism
Source: PLoS One. 2017 Aug 3;12(8):e0182328. doi: 10.1371/journal.pone.0182328 (PMC5542541; doi:10.1371/journal.pone.0182328)
Supplement: S5 Fig — (PDF) [file pone.0182328.s005.pdf]

S5 Fig

A

Transcript ID	Gene
Phvul.004G139700	Disease resistance protein (TIR-NBS-LRR class) family
Phvul.010G132200	Disease resistance protein (TIR-NBS-LRR class) family
Phvul.005G084100	Toll-Interleukin-Resistance (TIR) domain family protein
Phvul.010G027300	Disease resistance protein (TIR-NBS-LRR class) family
Phvul.004G140800	TIR-NBS-LRR class, putative transmembrane receptors; ATP binding
Phvul.004G140100	Disease resistance protein (TIR-NBS-LRR class), putative
Phvul.010G132000	Disease resistance protein (TIR-NBS-LRR class) family
Phvul.004G139800	TIR-NBS-LRR class, putative transmembrane receptors;ATP binding
Phvul.008G220600	Polyketide cyclase/dehydrase and lipid transport superfamily protein
Phvul.004G140700	Disease resistance protein (TIR-NBS-LRR class), putative
Phvul.010G054400	Disease resistance protein (TIR-NBS-LRR class) family
Phvul.003G109200	MLP-like protein 423
Phvul.010G101200	Disease resistance protein (TIR-NBS-LRR class), putative
Phvul.004G046400	Disease resistance protein (TIR-NBS-LRR class), putative
Phvul.010G136700	Disease resistance protein (TIR-NBS-LRR class), putative
Phvul.011G195800	Disease resistance protein (TIR-NBS-LRR class), putative
Phvul.010G025200	Disease resistance protein (TIR-NBS-LRR class) family
Phvul.001G093900	Disease resistance protein (TIR-NBS-LRR class), putative
Phvul.003G070900	Transmembrane receptors; ATP binding
Phvul.011G181400	Disease resistance protein (TIR-NBS-LRR class), putative
Phvul.010G065400	PYR1-like 4
Phvul.003G109000	MLP-like protein 423
Phvul.008G265900	Disease resistance protein (TIR-NBS-LRR class), putative
Phvul.010G026900	Disease resistance protein (TIR-NBS-LRR class) family
Phvul.008G195100	Disease resistance protein (TIR-NBS-LRR class), putative
Phvul.008G267700	Disease resistance protein (TIR-NBS-LRR class), putative
Phvul.010G026600	Disease resistance protein (TIR-NBS-LRR class) family
Phvul.011G140400	Disease resistance protein (TIR-NBS-LRR class), putative
Phvul.009G077200	PYR1-like 2

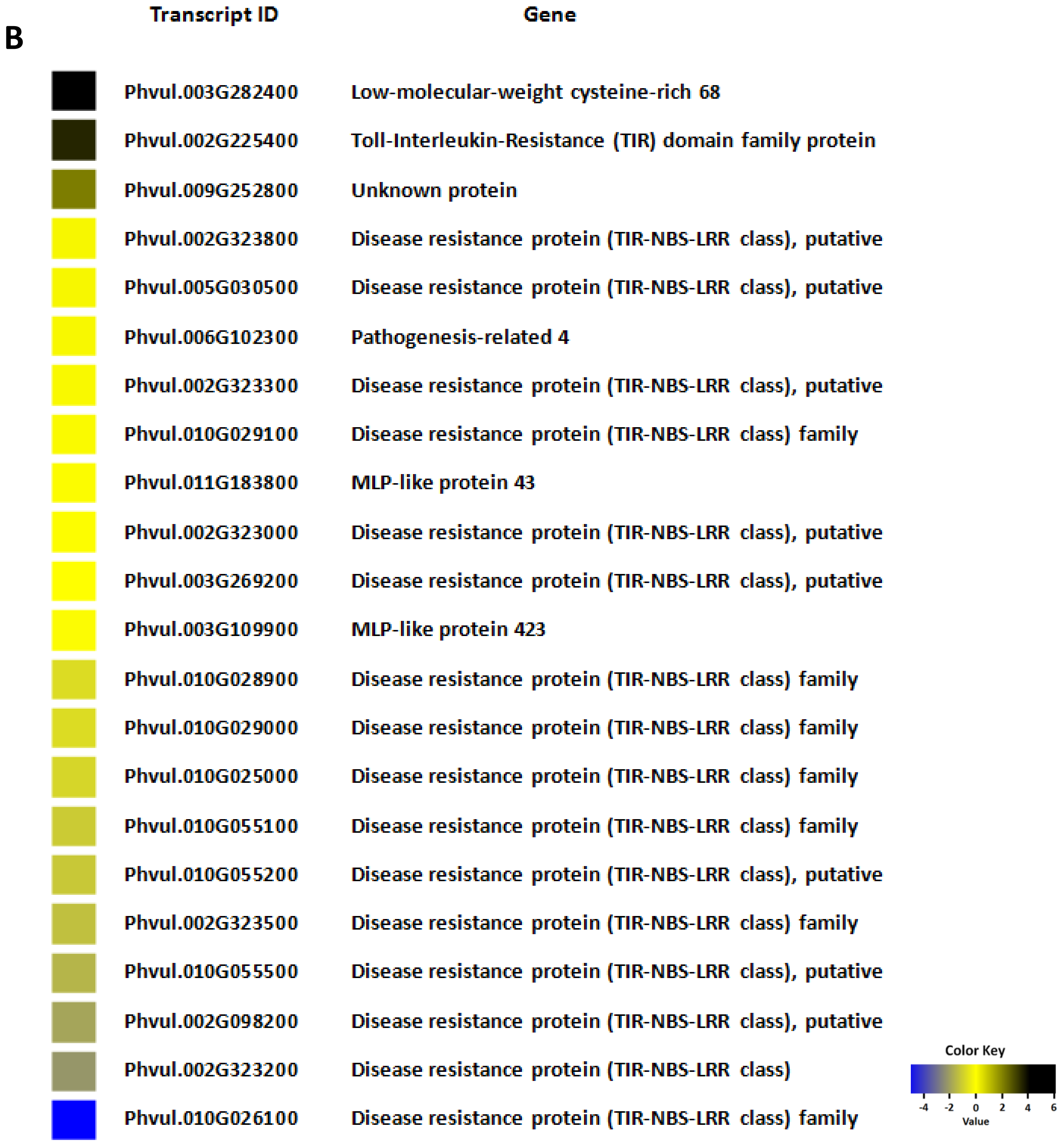

S5 Fig. DEGs of defense-responsive genes during root symbioses. Expression profile of unique defense-responsive genes in *P. vulgaris* roots colonized by (A) AMF and (B) rhizobia. Expression profile showing upregulated and downregulated DEGs obtained form GO analysis. Statistically significant DEGs were identified using an unpaired *t*-test (p<0.05) in symbiont treatment over controls (S3 Table). Fold-change values (over control) were used to plot heat maps. Color bar scale shows the fold-change range with red and green representing downregulation and upregulation, respectively.
