# Supplementary material for: Differentially expressed genes in mycorrhized and nodulated roots of common bean are associated with defense, cell wall architecture, N metabolism, and P metabolism
Source: PLoS One. 2017 Aug 3;12(8):e0182328. doi: 10.1371/journal.pone.0182328 (PMC5542541; doi:10.1371/journal.pone.0182328)
Supplement: S6 Fig — (PDF) [file pone.0182328.s006.pdf]

S6 Fig

A

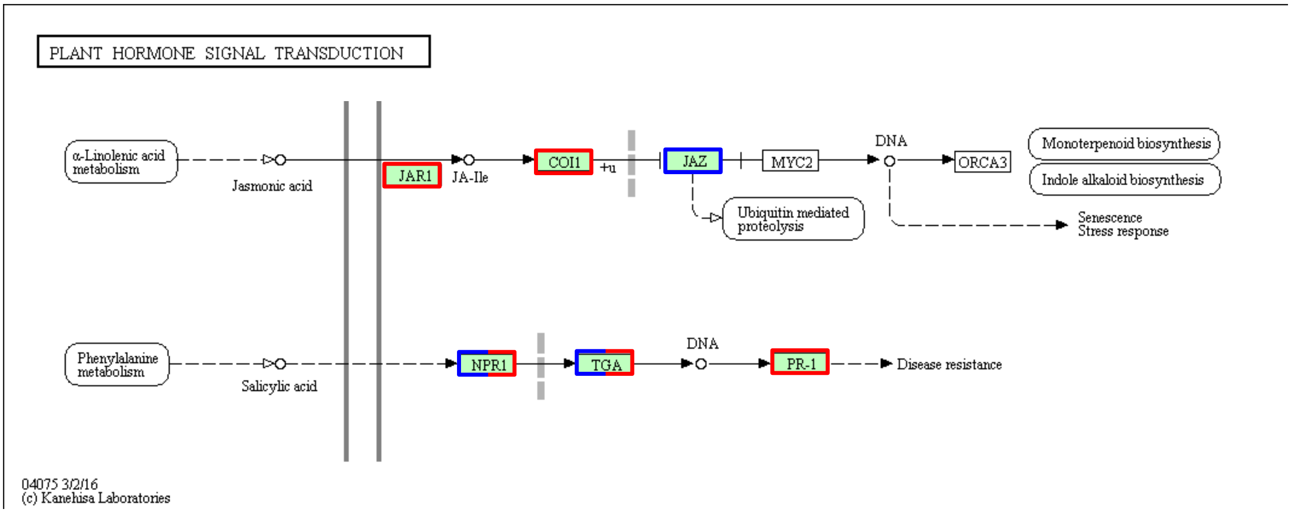

B

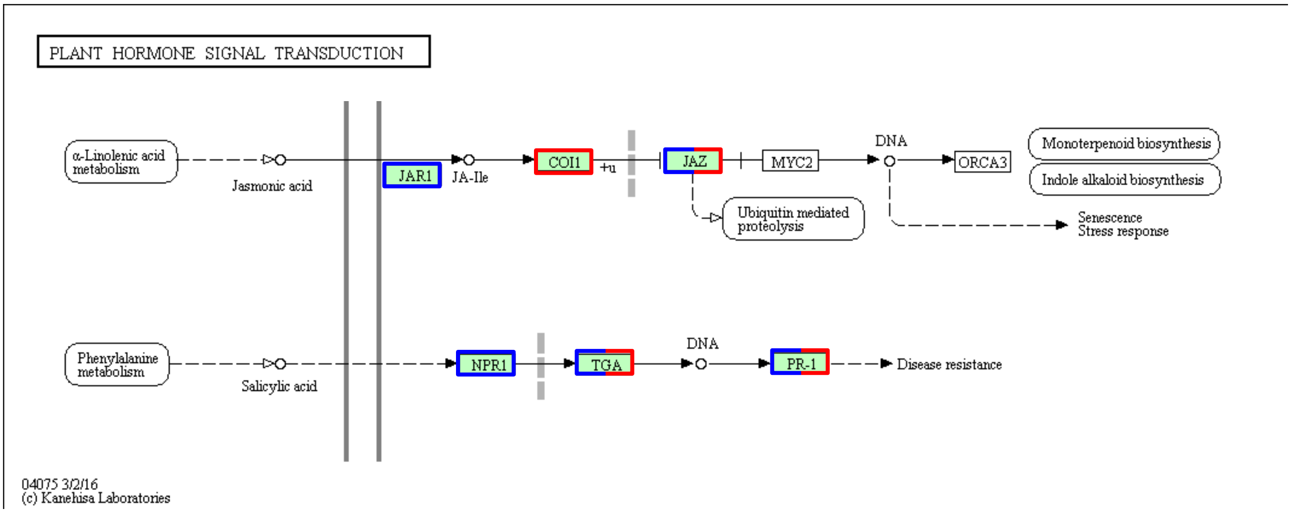

S6 Fig. Effect of root symbiosis on the plant signal transduction pathways in mycorrhized and nodulated *P. vulgaris* roots. The pathway model was obtained from KEGG Pathway [52] and modified. Differential expression patterns of key defense genes associated with jasmonic acid and salicylic acid signaling pathways in roots colonized by (A) AMF and (B) rhizobia. Blue and red borders surrounding EC identifiers represent genes that are upregulated and downregulated, respectively, relative to the controls. Borders with mixed colors indicate both upregulation and downregulation of different transcripts of the same gene.
