# Supplementary material for: Differentially expressed genes in mycorrhized and nodulated roots of common bean are associated with defense, cell wall architecture, N metabolism, and P metabolism
Source: PLoS One. 2017 Aug 3;12(8):e0182328. doi: 10.1371/journal.pone.0182328 (PMC5542541; doi:10.1371/journal.pone.0182328)
Supplement: S7 Fig — (PDF) [file pone.0182328.s007.pdf]

S7 Fig

A

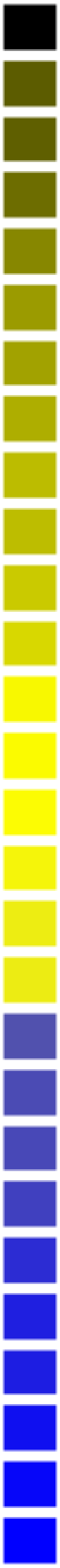

Transcript ID	Gene
Phvul.003G147700	Xyloglucan endotransglucosylase/hydrolase family protein
Phvul.003G147400	Xyloglucan endotransglycosylase 6
Phvul.005G011900	Plant invertase/pectin methylesterase inhibitor superfamily
Phvul.001G209100	Plant invertase/pectin methylesterase inhibitor superfamily
Phvul.003G137600	Xyloglucan endotransglycosylase 6
Phvul.003G052400	Xyloglucan:xyloglucosyl transferase 33
Phvul.003G147600	Xyloglucan endotransglucosylase/hydrolase family protein
Phvul.011G024800	Expansin-like A2
Phvul.003G015600	Pectin lyase-like superfamily protein
Phvul.009G203300	Reversibly glycosylated polypeptide 3
Phvul.007G210400	Plant invertase/pectin methylesterase inhibitor superfamily
Phvul.003G147500	Xyloglucan endotransglycosylase 6
Phvul.009G250600	Plant invertase/pectin methylesterase inhibitor superfamily
Phvul.010G123100	Pectin methylesterase 3
Phvul.011G117500	Expansin B3
Phvul.001G265300	Xyloglucan endotransglucosylase/hydrolase 15
Phvul.009G222100	Plant invertase/pectin methylesterase inhibitor superfamily
Phvul.007G212000	Pectin lyase-like superfamily protein
Phvul.011G189000	Root hair specific 12
Phvul.005G007800	Plant invertase/pectin methylesterase inhibitor superfamily
Phvul.003G213800	Expansin A18
Phvul.002G270900	Plant invertase/pectin methylesterase inhibitor superfamily
Phvul.009G222700	Plant invertase/pectin methylesterase inhibitor superfamily
Phvul.002G152900	Expansin A7
Phvul.007G198200	Plant invertase/pectin methylesterase inhibitor superfamily
Phvul.011G085200	Xyloglucan endotransglucosylase/hydrolase 32
Phvul.009G233200	Xyloglucan endotransglycosylase 6
Phvul.010G079900	Plant invertase/pectin methylesterase inhibitor superfamily

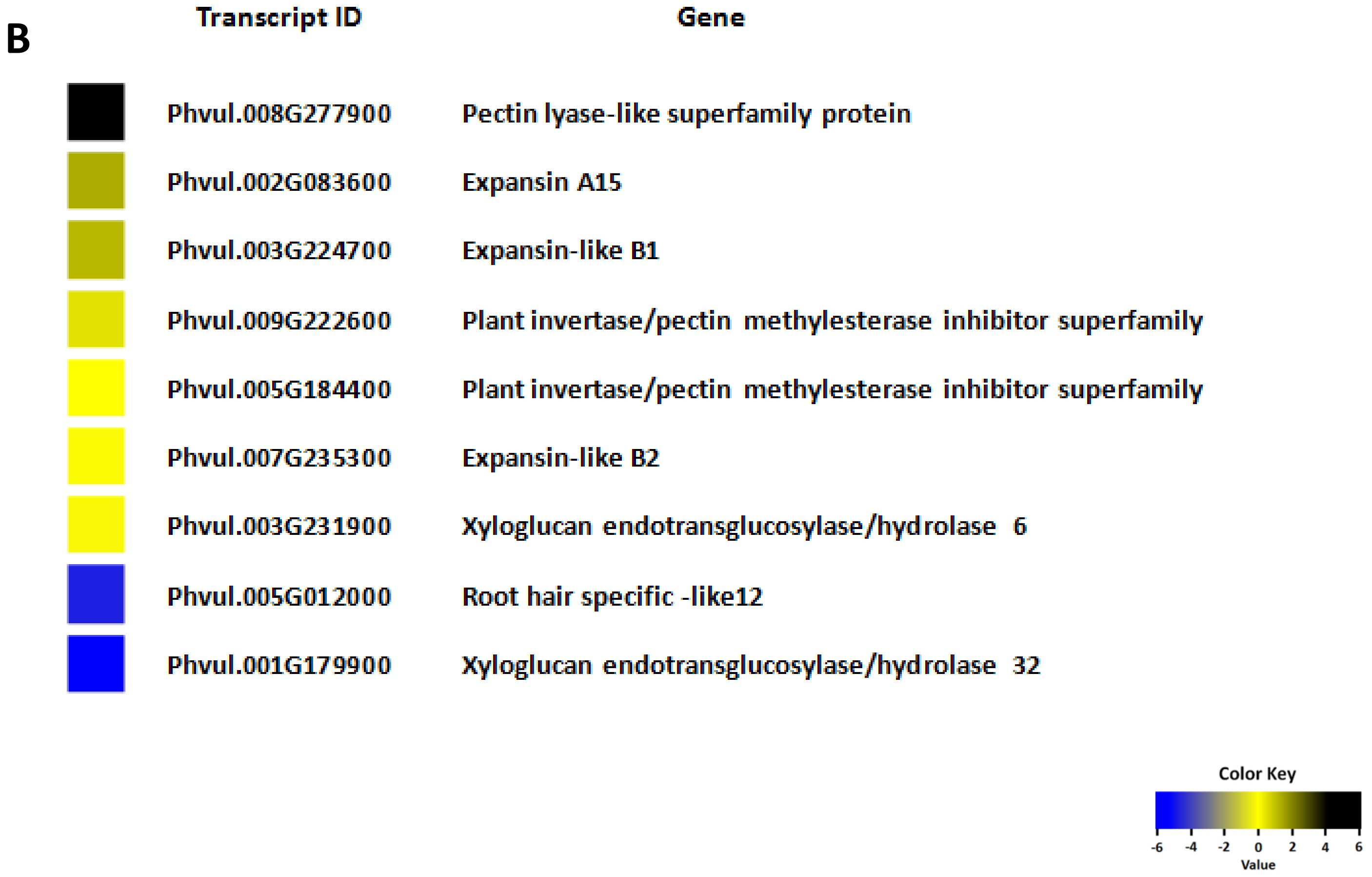

S7 Fig. DEGs of cell wall-related genes during root symbioses. Expression profile of unique cell wall-related genes in *P. vulgaris* roots colonized by (A) AMF and (B) rhizobia. Expression profile showing upregulated and downregulated DEGs obtained form GO analysis. Statistically significant DEGs were identified using an unpaired *t*-test (p<0.05), in symbiont treatment over controls (S3 Table). Fold-change values (over control) were used to plot heat maps. Color bar scale shows the fold-change range with red and green representing downregulation and upregulation, respectively.
