# Supplementary material for: Differentially expressed genes in mycorrhized and nodulated roots of common bean are associated with defense, cell wall architecture, N metabolism, and P metabolism
Source: PLoS One. 2017 Aug 3;12(8):e0182328. doi: 10.1371/journal.pone.0182328 (PMC5542541; doi:10.1371/journal.pone.0182328)
Supplement: S8 Fig — (PDF) [file pone.0182328.s008.pdf]

S8 Fig

A

Transcript ID	Gene
Phvul.002G267800	Integrase-type DNA-binding superfamily protein
Phvul.009G203400	AGAMOUS-like 8
Phvul.002G160100	WRKY DNA-binding protein 27
Phvul.001G192000	NAC domain containing protein 90
Phvul.011G119500	Basic helix-loop-helix (bHLH) DNA-binding superfamily protein
Phvul.004G046200	ATPase E1-E2 type family protein / haloacid dehalogenase-like hydrolase
Phvul.002G153900	Integrase-type DNA-binding superfamily protein
Phvul.003G212700	Integrase-type DNA-binding superfamily protein
Phvul.003G223600	Integrase-type DNA-binding superfamily protein
Phvul.006G204200	GTP cyclohydrolase II
Phvul.005G155800	Homolog of carrot EP3-3 chitinase
Phvul.002G256500	Signal responsive 1
Phvul.004G075500	NAC domain containing protein 41,NAC domain containing protein 83
Phvul.002G035100	Integrase-type DNA-binding superfamily protein
Phvul.010G057900	WRKY DNA-binding protein 70
Phvul.004G024700	AAA-ATPase 1
Phvul.003G268600	Basic chitinase
Phvul.010G015400	Delta 1-pyrroline-5-carboxylate synthase 2
Phvul.002G317400	ATPase E1-E2 type family protein / haloacid dehalogenase-like hydrolase
Phvul.011G014000	Cytokinin oxidase/dehydrogenase 6
Phvul.009G137500	WRKY family transcription factor
Phvul.010G117900	Basic helix-loop-helix (bHLH) DNA-binding family protein
Phvul.009G179600	Basic helix-loop-helix (bHLH) DNA-binding superfamily protein
Phvul.002G271000	Basic helix-loop-helix (bHLH) DNA-binding superfamily protein
Phvul.002G149500	DORNROSCHEN-like
Phvul.002G206300	Xylem NAC domain 1
Phvul.007G193400	Integrase-type DNA-binding superfamily protein
Phvul.003G235500	Calcium ATPase 2
Phvul.001G039900	WRKY DNA-binding protein 50
Phvul.001G187100	Integrase-type DNA-binding superfamily protein
Phvul.004G120500	Homeobox-leucine zipper family protein / lipid-binding START domain
Phvul.009G084400	Integrase-type DNA-binding superfamily protein
Phvul.003G153000	Class I glutamine amidotransferase-like superfamily protein
Phvul.003G116300	WRKY DNA-binding protein 48
Phvul.007G170200	Unknown protein
Phvul.006G179700	Integrase-type DNA-binding superfamily protein
Phvul.003G111800	AP2/B3 transcription factor family protein
Phvul.001G216600	S-adenosylmethionine synthetase 2
Phvul.003G167700	Auxin-induced protein 13
Phvul.002G019100	Heat shock factor 4
Phvul.003G119800	Calmodulin-binding transcription activator with CG-1 and Ankyrin domains
Phvul.005G181800	WRKY DNA-binding protein 40
Phvul.003G140800	Basic helix-loop-helix (bHLH) DNA-binding superfamily protein
Phvul.006G188900	NAC domain containing protein 25
Phvul.007G272900	Integrase-type DNA-binding superfamily protein
Phvul.004G042400	AGAMOUS-like 8
Phvul.002G185800	Glutamate decarboxylase
Phvul.006G169600	K-box region and MADS-box transcription factor family protein
Phvul.009G235700	Basic helix-loop-helix (bHLH) DNA-binding superfamily protein
Phvul.006G179800	Ethylene responsive element binding factor 2

Transcript ID	Gene
Phvul.010G148700	HD-ZIP IV family of homeobox-leucine zipper protein with lipid START domain
Phvul.007G066500	Integrase-type DNA-binding superfamily protein
Phvul.009G152800	NAC domain containing protein 102
Phvul.001G044500	Integrase-type DNA-binding superfamily protein
Phvul.003G070300	Homeobox 1
Phvul.002G036000	Integrase-type DNA-binding superfamily protein
Phvul.006G181500	NAC domain containing protein 73
Phvul.008G194600	NAC domain containing protein 42
Phvul.001G062100	Dof-type zinc finger DNA-binding family protein
Phvul.009G137600	Sigma factor E
Phvul.009G190200	Homeobox 1
Phvul.004G083100	GATA transcription factor 4
Phvul.003G292400	Redox responsive transcription factor 1
Phvul.001G164900	Auxin-induced protein 13
Phvul.002G011400	Calcium ATPase 2
Phvul.002G196800	WRKY DNA-binding protein 28
Phvul.008G037900	NAC domain containing protein 100
Phvul.001G120200	Transcription elongation factor (TFIIS) family protein
Phvul.001G214400	WRKY DNA-binding protein 23
Phvul.009G231600	Basic helix-loop-helix (bHLH) DNA-binding family protein
Phvul.001G216700	S-adenosylmethionine synthetase 1
Phvul.005G011600	ABA-responsive element binding protein 3
Phvul.001G084000	Integrase-type DNA-binding superfamily protein
Phvul.011G101900	WRKY family transcription factor
Phvul.001G106400	Cycling DOF factor 3
Phvul.002G007500	Basic helix-loop-helix (bHLH) DNA-binding superfamily protein
Phvul.004G004900	NAC (No Apical Meristem) domain transcriptional regulator superfamily protein
Phvul.002G307000	NAC domain containing protein 75
Phvul.001G247200	Arogenate dehydrogenase
Phvul.009G123300	Integrase-type DNA-binding superfamily protein
Phvul.002G260400	Endoplasmic reticulum-type calcium-transporting ATPase 3
Phvul.009G039000	NAC transcription factor-like 9
Phvul.002G163700	Integrase-type DNA-binding superfamily protein
Phvul.006G119100	WRKY DNA-binding protein 48
Phvul.002G316500	NAC domain containing protein 58
Phvul.002G265400	WRKY DNA-binding protein 51
Phvul.005G138900	Phototropin 1
Phvul.004G169400	AP2/B3-like transcriptional factor family protein
Phvul.007G223400	Heat shock transcription factor A3
Phvul.002G186000	Glutamate decarboxylase
Phvul.006G047300	WRKY family transcription factor
Phvul.006G110200	Cytokinin response factor 4
Phvul.010G070200	HSI2-like 1
Phvul.007G148000	Methionine adenosyltransferase 3
Phvul.003G231200	Basic helix-loop-helix (bHLH) DNA-binding superfamily protein
Phvul.001G206100	Thiamin pyrophosphokinase1
Phvul.006G114000	ABA-inducible BHLH-type transcription factor
Phvul.006G074600	WRKY DNA-binding protein 33
Phvul.009G138600	WRKY DNA-binding protein 13
Phvul.002G185900	Glutamate decarboxylase

Transcript ID	Gene
Phvul.008G115100	Delta 1-pyrroline-5-carboxylate synthase 2
Phvul.004G076900	Arabidopsis NAC domain containing protein 87
Phvul.002G187900	Homeodomain GLABROUS 12
Phvul.007G224800	Homeobox 51
Phvul.007G102800	AP2/B3 transcription factor family protein
Phvul.005G173000	AUX/IAA transcriptional regulator family protein
Phvul.002G215500	AGAMOUS-like 21
Phvul.002G114100	Basic chitinase
Phvul.007G176700	AUX/IAA transcriptional regulator family protein
Phvul.009G136400	Dof-type zinc finger DNA-binding family protein
Phvul.007G135900	Integrase-type DNA-binding superfamily protein
Phvul.002G055700	Ethylene responsive element binding factor 1
Phvul.009G037300	K-box region and MADS-box transcription factor family protein
Phvul.008G176200	Class-II DAHP synthetase family protein
Phvul.002G160000	Sporulation 11-2
Phvul.001G100500	Arabidopsis NAC domain containing protein 87
Phvul.011G024700	NAC domain containing protein 36
Phvul.010G050900	H(+)-ATPase 11
Phvul.008G098900	Related to AP2 1
Phvul.003G242900	Basic helix-loop-helix (bHLH) DNA-binding superfamily protein
Phvul.005G181300	Basic helix-loop-helix (bHLH) DNA-binding superfamily protein
Phvul.005G084500	NAC (No Apical Meristem) domain transcriptional regulator superfamily protein
Phvul.008G027900	K-box region and MADS-box transcription factor family protein
Phvul.003G027100	NAC (No Apical Meristem) domain transcriptional regulator superfamily protein
Phvul.009G047500	Dof-type zinc finger DNA-binding family protein
Phvul.001G128800	Cytokinin oxidase/dehydrogenase 1
Phvul.007G095000	Auxin response factor 16
Phvul.007G095300	GATA transcription factor 4
Phvul.006G087600	Glutamate decarboxylase
Phvul.002G209300	Calmodulin-binding transcription activator protein with CG-1 and Ankyrin domains
Phvul.010G114800	Basic helix-loop-helix (bHLH) DNA-binding superfamily protein
Phvul.007G056600	Amine oxidase 1
Phvul.009G186000	NAC domain containing protein 100
Phvul.002G035900	Integrase-type DNA-binding superfamily protein
Phvul.005G116000	WRKY family transcription factor
Phvul.003G107900	Ethylene-responsive element binding protein
Phvul.003G026800	Basic helix-loop-helix (bHLH) DNA-binding superfamily protein
Phvul.001G034200	Homeobox gene 1
Phvul.009G099600	N-acetyl-l-glutamate kinase
Phvul.001G226500	GATA transcription factor 16
Phvul.009G065800	Heat shock transcription factor B4
Phvul.008G019600	Related to AP2 6l
Phvul.006G093900	Basic helix-loop-helix (bHLH) DNA-binding superfamily protein
Phvul.001G121200	Basic helix-loop-helix (bHLH) DNA-binding superfamily protein
Phvul.009G110400	GATA transcription factor 12
Phvul.001G154700	Heat shock transcription factor A6B
Phvul.007G278200	Heat shock transcription factor A6B
Phvul.008G169100	ABA-responsive element binding protein 3
Phvul.002G254500	Integrase-type DNA-binding superfamily protein
Phvul.004G044100	Heat shock protein 101

Transcript ID	Gene
Phvul.009G239100	LYSYL-TRNA synthetase 1
Phvul.009G204000	OBF binding protein 4
Phvul.008G202500	Heat shock transcription factor B4
Phvul.005G048700	Aldolase-type TIM barrel family protein
Phvul.001G218600	Chitinase family protein
Phvul.007G208500	Phytochrome interacting factor 3
Phvul.009G026400	Serine hydroxymethyltransferase 4
Phvul.005G170600	Integrase-type DNA-binding superfamily protein
Phvul.007G019300	Hydroxypyruvate reductase
Phvul.008G222700	Basic helix-loop-helix (bHLH) DNA-binding superfamily protein
Phvul.007G271700	Signal transduction histidine kinase, hybrid-type, ethylene sensor
Phvul.002G240900	WRKY DNA-binding protein 65
Phvul.003G180000	Related to AP2 11
Phvul.003G098300	tRNA/rRNA methyltransferase (SpoU) family protein
Phvul.002G064100	KNOTTED1-like homeobox gene 6
Phvul.011G177100	Hydroxymethylbilane synthase
Phvul.002G249900	6-phosphogluconate dehydrogenase family protein
Phvul.008G290000	AAA-type ATPase family protein
Phvul.001G213600	WRKY DNA-binding protein 69
Phvul.009G228000	Growth-regulating factor 5
Phvul.008G144300	Basic helix-loop-helix (bHLH) DNA-binding superfamily protein
Phvul.010G146600	Integrase-type DNA-binding superfamily protein
Phvul.003G189600	Pyridine nucleotide-disulphide oxidoreductase family protein
Phvul.003G106200	Copper amine oxidase family protein
Phvul.006G101700	bZIP transcription factor family protein
Phvul.008G184800	beta HLH protein 93
Phvul.007G135300	Integrase-type DNA-binding superfamily protein
Phvul.001G254000	Homeobox protein 16
Phvul.009G018700	ABA-responsive element binding protein 3
Phvul.008G042200	Homeobox 3
Phvul.007G241600	Integrase-type DNA-binding superfamily protein
Phvul.006G015700	Heat shock transcription factor B4
Phvul.002G316900	BR enhanced expression 3
Phvul.010G153200	DNA photolyase family protein
Phvul.004G043900	Heat shock protein 101
Phvul.009G208600	Unknown protein
Phvul.004G045800	Basic leucine-zipper 42
Phvul.001G022900	Heat shock transcription factor B4
Phvul.003G233200	Basic leucine-zipper 5
Phvul.010G092300	Related to AP2 11
Phvul.001G160100	Integrase-type DNA-binding superfamily protein
Phvul.009G102000	FAD-dependent oxidoreductase family protein
Phvul.009G002200	GLN phosphoribosyl pyrophosphate amidotransferase 1
Phvul.002G030800	GATA type zinc finger transcription factor family protein
Phvul.007G032200	Indole-3-acetic acid inducible 32
Phvul.003G222600	Integrase-type DNA-binding superfamily protein
Phvul.003G097900	Adenosylmethionine decarboxylase family protein
Phvul.006G149700	ATPase, F1 complex, gamma subunit protein
Phvul.003G264600	Histidine kinase 1
Phvul.009G075100	Branched-chain amino acid transaminase 2
Phvul.005G052500	Nicotianamine synthase 4

B

Transcript ID	Gene
Phvul.003G136400	Cytokinin oxidase 3
Phvul.008G237400	Glutamine synthase clone R1
Phvul.002G068500	Werner syndrome-like exonuclease
Phvul.009G231800	Cytokinin oxidase 2
Phvul.011G064900	WUSCHEL related homeobox 2
Phvul.007G048000	AGAMOUS-like 62
Phvul.009G196900	Integrase-type DNA-binding superfamily protein
Phvul.003G028800	bZIP transcription factor family protein
Phvul.007G028100	Isopentenyltransferase 1
Phvul.009G260100	AP2/B3-like transcriptional factor family protein
Phvul.009G145800	Beta HLH protein 93
Phvul.003G068700	WRKY DNA-binding protein 72
Phvul.006G065300	ThiaminC
Phvul.003G268500	Basic chitinase
Phvul.009G170600	BTB and TAZ domain protein 2
Phvul.009G077400	Glycine decarboxylase P-protein 2
Phvul.008G043500	Integrase-type DNA-binding superfamily protein
Phvul.002G229800	AP2/B3-like transcriptional factor family protein
Phvul.006G173500	Related to AP2 11
Phvul.002G048200	Protodermal factor 2
Phvul.008G045100	Homeobox-leucine zipper protein 3
Phvul.010G120900	AP2/B3-like transcriptional factor family protein
Phvul.001G229500	Glutamine synthetase
Phvul.007G234300	Uricase
Phvul.002G329800	Beta-6 tubulin
Phvul.006G013200	Homeobox protein 6
Phvul.008G129500	Dicer-like 2
Phvul.003G182700	K-box region and MADS-box transcription factor family protein
Phvul.007G019000	Basic helix-loop-helix (bHLH) DNA-binding superfamily protein
Phvul.006G069300	Glutamine-dependent asparagine synthase 1
Phvul.007G086600	Integrase-type DNA-binding superfamily protein
Phvul.009G127000	Response regulator 9
Phvul.003G207500	AAA-type ATPase family protein
Phvul.006G145800	KNOX/ELK homeobox transcription factor
Phvul.002G089700	WRKY DNA-binding protein 9
Phvul.005G114600	Homeodomain GLABROUS 2
Phvul.003G124600	Indole-3-acetic acid inducible 31
Phvul.009G232700	GATA type zinc finger transcription factor family protein
Phvul.009G033800	Basic helix-loop-helix (bHLH) DNA-binding superfamily protein
Phvul.009G116700	Basic chitinase
Phvul.009G060200	Cytokinin oxidase 5
Phvul.008G181500	Basic helix-loop-helix (bHLH) DNA-binding superfamily protein
Phvul.009G013900	K-box region and MADS-box transcription factor family protein
Phvul.005G027100	Response regulator 9
Phvul.011G157500	Tyrosine transaminase family protein
Phvul.006G105100	Zinc ion binding;DNA binding
Phvul.008G185100	Glutamyl-tRNA reductase family protein
Phvul.001G193100	AP2/B3-like transcriptional factor family protein
Phvul.003G250500	BEL1-like homeodomain 4
Phvul.002G118900	hydroxy methylglutaryl CoA reductase 1

Transcript ID	Gene
Phvul.001G218800	Phytochrome interacting factor 3
Phvul.010G050800	Ethylene-responsive element binding factor 13
Phvul.011G070300	Phototropin 1
Phvul.006G186700	Allantoinase
Phvul.002G142800	Beta HLH protein 93
Phvul.003G032800	Histone acetyltransferase of the CBP family 12
Phvul.001G111800	Related to AP2 11
Phvul.002G007900	Tubulin alpha-2 chain
Phvul.009G004800	BEL1-like homeodomain 6
Phvul.007G056500	Copper amine oxidase family protein
Phvul.003G284500	Basic helix-loop-helix (bHLH) DNA-binding superfamily protein
Phvul.002G216700	Basic helix-loop-helix (bHLH) DNA-binding superfamily protein
Phvul.009G002200	Phosphoribosyl pyrophosphate amidotransferase
Phvul.002G027200	Homeobox protein 40
Phvul.006G155800	Glutamine synthetase 2
Phvul.005G148000	Xanthine dehydrogenase 1
Phvul.011G180500	DNA glycosylase superfamily protein
Phvul.002G088400	Basic helix-loop-helix (bHLH) DNA-binding superfamily protein
Phvul.008G004200	Glycine ligase
Phvul.002G273500	Phosphoribosylaminoimidazole carboxylase
Phvul.011G050500	AP2/B3-like transcriptional factor family protein
Phvul.008G289900	Coproporphyrinogen III oxidase
Phvul.003G030500	Endonuclease 2
Phvul.009G116500	Basic chitinase
Phvul.003G286400	beta HLH protein 93
Phvul.007G019500	AP2/B3-like transcriptional factor family protein
Phvul.001G156600	Indole-3-acetic acid inducible 14
Phvul.004G047600	G-box binding factor 4
Phvul.009G053900	NADH-dependent glutamate synthase 2 (GOGAT)
Phvul.004G070100	Aspartate kinase family protein,aspartate kinase 1
Phvul.005G161900	Basic helix-loop-helix (bHLH) DNA-binding superfamily protein
Phvul.002G056300	Homeobox-leucine zipper protein 4 (HB-4) / HD-ZIP protein
Phvul.010G011900	POX (plant homeobox) family protein
Phvul.002G322300	Response regulator 14
Phvul.008G270500	WRKY family transcription factor
Phvul.005G044600	NAC (No Apical Meristem) domain transcriptional regulator superfamily protein
Phvul.002G112200	K-box region and MADS-box transcription factor family protein
Phvul.007G208900	Chitinase family protein
Phvul.002G070300	Domains rearranged methylase 1
Phvul.007G272800	Integrase-type DNA-binding superfamily protein
Phvul.002G161000	P-loop containing nucleoside triphosphate hydrolases superfamily protein
Phvul.002G283600	NAC domain containing protein 83
Phvul.011G091400	C-repeat/DRE binding factor 2
Phvul.006G114900	Dof-type zinc finger DNA-binding family protein
Phvul.002G052100	Basic helix-loop-helix (bHLH) DNA-binding superfamily protein
Phvul.003G035100	DNA glycosylase superfamily protein
Phvul.011G003900	NAC domain containing protein 70
Phvul.003G159200	H(+)-ATPase 8
Phvul.001G156500	AUX/IAA transcriptional regulator family protein
Phvul.001G099400	DEAD/DEAH box helicase, putative

Transcript ID	Gene
Phvul.002G309200	AGAMOUS-like 29
Phvul.006G015500	P-loop containing nucleoside triphosphate hydrolases superfamily protein
Phvul.008G122100	AP2/B3-like transcriptional factor family protein
Phvul.002G061000	NAC 007
Phvul.003G251800	Homeobox protein 40
Phvul.005G041900	Cofactor of nitrate reductase and xanthine dehydrogenase 2
Phvul.009G077500	Cytokinin-responsive gata factor 1
Phvul.002G110600	Basic leucine-zipper 42
Phvul.009G162000	Basic helix-loop-helix (bHLH) DNA-binding superfamily protein
Phvul.005G105200	Integrase-type DNA-binding superfamily protein
Phvul.008G284200	Phytochromobilin:ferredoxin oxidoreductase, phytochromobilin synthase (HY2)
Phvul.001G032500	Pseudouridine synthase family protein
Phvul.009G260800	Autoinhibited H(+)-ATPase isoform 10
Phvul.001G126400	Basic helix-loop-helix (bHLH) DNA-binding superfamily protein
Phvul.002G176000	DEAD/DEAH box RNA helicase family protein
Phvul.008G070300	Ribosomal RNA adenine dimethylase family protein
Phvul.003G007500	AP2/B3-like transcriptional factor family protein
Phvul.002G309500	AGAMOUS-like 91
Phvul.003G040500	Copper amine oxidase family protein
Phvul.005G140700	DHBP synthase RibB-like alpha/beta domain;GTP cyclohydrolase II
Phvul.001G173700	WRKY family transcription factor family protein
Phvul.009G065700	Basic helix-loop-helix (bHLH) DNA-binding superfamily protein
Phvul.001G106700	WUSCHEL related homeobox 11
Phvul.009G176700	KNOTTED-like from Arabidopsis thaliana
Phvul.010G158300	Basic helix-loop-helix (bHLH) DNA-binding superfamily protein
Phvul.003G182800	K-box region and MADS-box transcription factor family protein
Phvul.010G120700	NAC transcription factor-like 9
Phvul.005G080400	WRKY DNA-binding protein 70
Phvul.002G270500	Amino acid kinase family protein
Phvul.002G100800	Transcriptional factor B3 family protein
Phvul.006G106100	Ethylene response factor 1
Phvul.007G215000	Signal transduction histidine kinase
Phvul.001G160200	Ethylene response factor 1
Phvul.009G213800	MADS-box transcription factor family protein
Phvul.003G153900	DNA/RNA polymerases superfamily protein
Phvul.001G196100	Dof-type zinc finger DNA-binding family protein
Phvul.006G202200	K-box region and MADS-box transcription factor family protein
Phvul.010G151700	Indole-3-acetic acid inducible 19
Phvul.010G018200	HY5-homolog
Phvul.008G041800	Basic helix-loop-helix (bHLH) DNA-binding family protein
Phvul.009G108300	Phosphorylase superfamily protein
Phvul.004G143100	NAC domain containing protein 57
Phvul.010G131000	DNA-directed RNA polymerase family protein
Phvul.010G109700	Glyoxylate reductase 1
Phvul.010G045900	Paxneb protein-related
Phvul.010G088100	AGAMOUS-like 14
Phvul.006G196600	Basic helix-loop-helix (bHLH) DNA-binding superfamily protein
Phvul.008G203400	TATA binding protein associated factor 21kDa subunit
Phvul.008G239700	Glutamate decarboxylase
Phvul.005G168900	HD-ZIP IV family of homeobox-leucine zipper protein with lipid START domain

Transcript ID	Gene
Phvul.008G236100	RNA polymerase subunit beta
Phvul.003G132000	RNA polymerase III RPC4
Phvul.007G229600	Basic-leucine zipper (bZIP) transcription factor family protein
Phvul.007G089600	Vascular related NAC-domain protein 7
Phvul.007G208800	Indole-3-acetic acid inducible 30
Phvul.009G169300	AP2/B3-like transcriptional factor family protein
Phvul.008G188200	Ribonuclease P family protein / Rpp14 family protein
Phvul.004G077400	NAC domain containing protein 47
Phvul.009G080000	WRKY DNA-binding protein 50
Phvul.002G085700	No Apical Meristem domain transcriptional regulator superfamily protein
Phvul.002G266400	WRKY DNA-binding protein 13
Phvul.005G152500	Endonuclease III 2
Phvul.001G050500	Adenine phosphoribosyl transferase 2
Phvul.003G274800	Phototropin 2
Phvul.002G143900	AGAMOUS-like 21
Phvul.005G047300	NAC domain containing protein 20
Phvul.003G037100	DNA glycosylase superfamily protein
Phvul.006G127100	Dicer-like 2
Phvul.001G106000	Cyclin/Brf1-like TBP-binding protein
Phvul.002G305000	1-amino-cyclopropane-1-carboxylate synthase 7
Phvul.010G073400	APRATAXIN-like
Phvul.005G007900	NAC 007

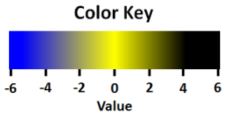

S8 Fig. DEGs of N metabolism genes during root symbioses. Expression profile of unique N metabolism genes in *P. vulgaris* roots colonized by (A) AMF and (B) rhizobia. Expression profile showing upregulated and downregulated DEGs obtained form GO analysis. Statistically significant DEGs were identified using an unpaired *t*-test ($p<0.05$), in symbiont treatment over controls (S3 Table). Fold-change values (over control) were used to plot heat maps. Color bar scale shows the fold-change range with red and green representing downregulation and upregulation, respectively.
