# Supplementary material for: Differentially expressed genes in mycorrhized and nodulated roots of common bean are associated with defense, cell wall architecture, N metabolism, and P metabolism
Source: PLoS One. 2017 Aug 3;12(8):e0182328. doi: 10.1371/journal.pone.0182328 (PMC5542541; doi:10.1371/journal.pone.0182328)
Supplement: S9 Fig — (PDF) [file pone.0182328.s009.pdf]

S9 Fig

A

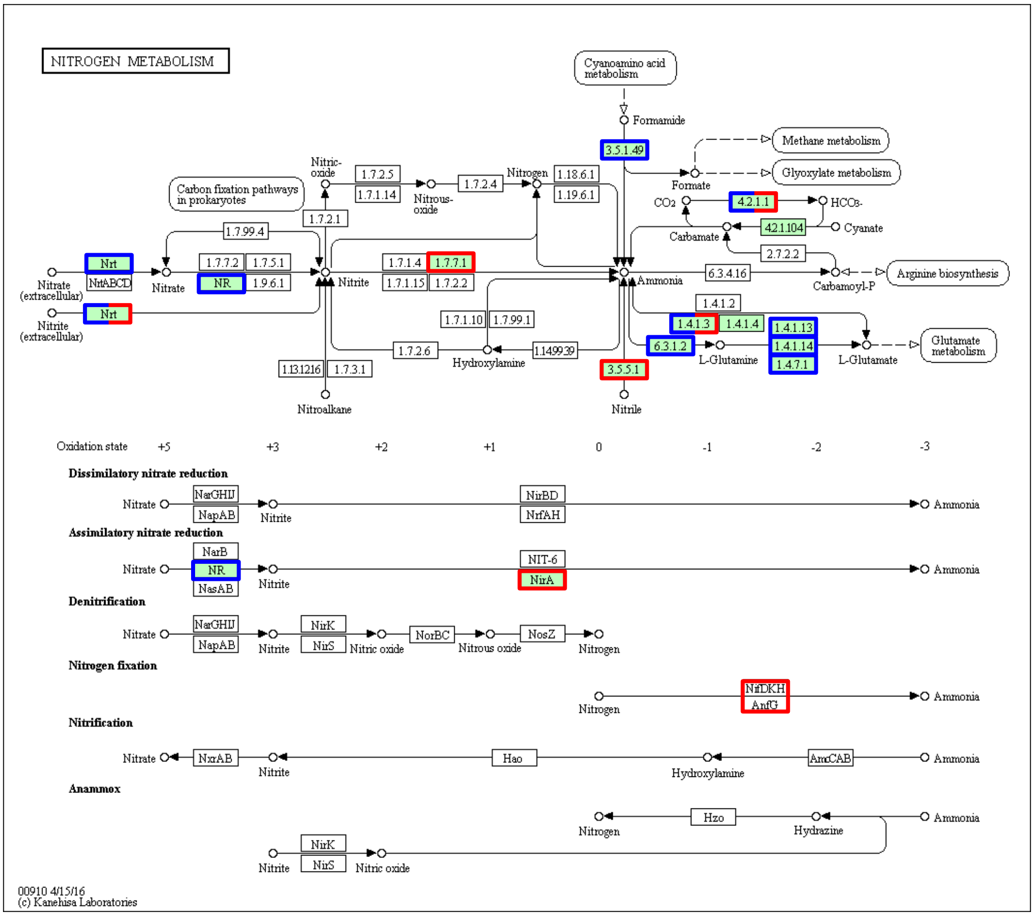

NITROGEN METABOLISM
Cyanoamino acid metabolism
Formamide
3.5.1.49
Methane metabolism
Glyoxylate metabolism
Formate
CO2
4.2.1.1
HCO3-
4.2.1.104
Cyanate
Carbamate
2.7.2.2
Carbamoyl-P
Arginine biosynthesis
6.3.4.16
Ammonia
Nitric-oxide
1.7.2.5
1.7.1.14
Nitrous-oxide
1.7.2.4
Nitrogen
1.18.6.1
1.19.6.1
Carbon fixation pathways in prokaryotes
1.7.2.1
1.7.99.4
Nrt
NrtABCD
Nitrate (extracellular)
Nitrate
1.7.7.2
1.7.5.1
NR
1.9.6.1
Nitrite
1.7.1.4
1.7.7.1
1.7.1.15
1.7.2.2
Nitrite (extracellular)
1.4.1.2
1.4.1.3
1.4.1.4
1.4.1.13
1.4.1.14
1.4.7.1
6.3.1.2
L-Glutamine
L-Glutamate
Glutamate metabolism
1.7.1.10
1.7.99.1
1.7.2.6
Hydroxylamine
1.14.99.39
1.13.12.16
1.7.3.1
Nitroalkane
3.5.5.1
Nitrile
Oxidation state +5 +3 +2 +1 0 -1 -2 -3
Dissimilatory nitrate reduction
Nitrate NarGHIJ NapAB Nitrite NirBD NrfAH Ammonia
Assimilatory nitrate reduction
Nitrate NarB NR NasAB Nitrite NIT-6 NirA Ammonia
Denitrification
Nitrate NarGHIJ NapAB Nitrite NirK NirS Nitric oxide NorBC Nitrous oxide NosZ Nitrogen
Nitrogen fixation
Nitrogen NifDKH AnfG Ammonia
Nitrification
Nitrate NxrAB Nitrite Hao Hydroxylamine AmoCAB Ammonia
Anammox
Nitrogen Hzo Hydrazine Ammonia
Nitrite NirK NirS Nitric oxide
00910 4/15/16
(c) Kanehisa Laboratories

B

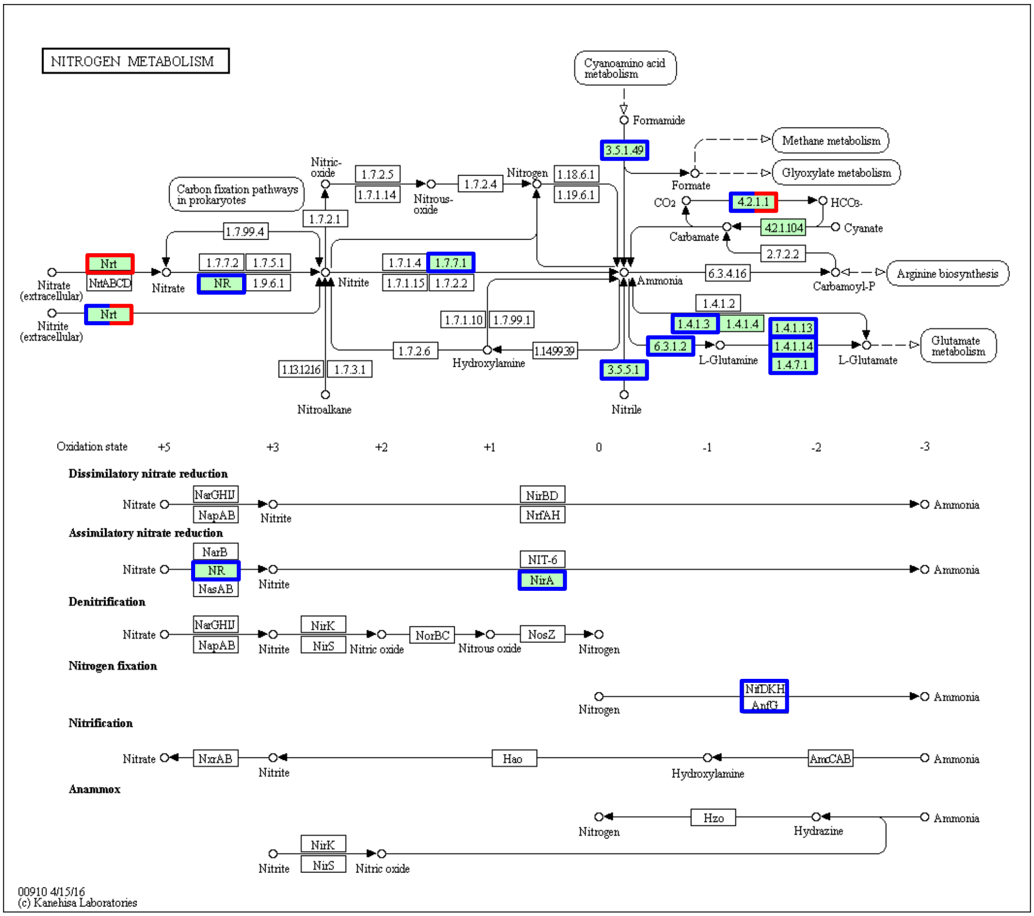

NITROGEN METABOLISM
Cyanoamino acid metabolism
Formamide
3.5.1.49
Methane metabolism
Glyoxylate metabolism
Formate
CO2
4.2.1.1
HCO3-
4.2.1.104
Cyanate
Carbamate
2.7.2.2
Carbamoyl-P
Arginine biosynthesis
6.3.4.16
Ammonia
Nitric-oxide
1.7.2.5
1.7.1.14
Nitrous-oxide
1.7.2.4
Nitrogen
1.18.6.1
1.19.6.1
Carbon fixation pathways in prokaryotes
1.7.2.1
1.7.99.4
Nrt
NrtABCD
Nitrate (extracellular)
Nitrate
1.7.7.2
1.7.5.1
NR
1.9.6.1
Nitrite
1.7.1.4
1.7.7.1
1.7.1.15
1.7.2.2
Nitrite (extracellular)
1.4.1.2
1.4.1.3
1.4.1.4
1.4.1.13
1.4.1.14
1.4.7.1
6.3.1.2
L-Glutamine
L-Glutamate
Glutamate metabolism
1.7.1.10
1.7.99.1
1.7.2.6
Hydroxylamine
1.14.99.39
1.13.12.16
1.7.3.1
Nitroalkane
3.5.5.1
Nitrile
Oxidation state +5 +3 +2 +1 0 -1 -2 -3
Dissimilatory nitrate reduction
Nitrate NarGHIJ NapAB Nitrite NirBD NrfAH Ammonia
Assimilatory nitrate reduction
Nitrate NarB NR NasAB Nitrite NIT-6 NirA Ammonia
Denitrification
Nitrate NarGHIJ NapAB Nitrite NirK NirS Nitric oxide NorBC Nitrous oxide NosZ Nitrogen
Nitrogen fixation
Nitrogen NifDKH AnfG Ammonia
Nitrification
Nitrate NxrAB Nitrite Hao Hydroxylamine AmoCAB Ammonia
Anammox
Nitrogen Hzo Hydrazine Ammonia
Nitrite NirK NirS Nitric oxide
00910 4/15/16
(c) Kanehisa Laboratories

S9 Fig. Effect of root symbiosis on the nitrogen metabolism pathway in mycorrhized and nodulated roots. The pathway model was obtained from KEGG Pathway [52]. Differential expression patterns of key genes and enzymes of nitrogen metabolism in *P. vulgaris* roots colonized by (A) AMF and (B) rhizobia. Blue and red borders surrounding EC identifiers represent genes that were upregulated and downregulated, respectively, relative to the controls. Borders with mixed colors indicate both upregulation and downregulation of different transcripts of the same gene.
