# Supplementary material for: Differentially expressed genes in mycorrhized and nodulated roots of common bean are associated with defense, cell wall architecture, N metabolism, and P metabolism
Source: PLoS One. 2017 Aug 3;12(8):e0182328. doi: 10.1371/journal.pone.0182328 (PMC5542541; doi:10.1371/journal.pone.0182328)
Supplement: S10 Fig — (PDF) [file pone.0182328.s010.pdf]

S10 Fig

A

Transcript ID	Gene
Phvul.002G123500	Leucine-rich repeat protein kinase family protein
Phvul.010G127500	Leucine-rich repeat protein kinase family protein
Phvul.008G174900	Protein kinase superfamily protein
Phvul.006G212500	MAPK/ERK kinase kinase 1
Phvul.002G196200	Leucine-rich receptor-like protein kinase family protein
Phvul.008G029500	Malectin/receptor-like protein kinase family protein
Phvul.003G158600	S-locus lectin protein kinase family protein
Phvul.004G155500	PR5-like receptor kinase
Phvul.007G048700	Cystein-rich RLK (RECEPTOR-like protein kinase) 29
Phvul.003G062600	Cystein-rich RLK (RECEPTOR-like protein kinase) 2
Phvul.001G076100	Protein kinase superfamily protein
Phvul.003G154000	Leucine-rich receptor-like protein kinase family protein
Phvul.011G155800	Diacylglycerol kinase 5
Phvul.008G047800	Leucine-rich repeat receptor-like protein kinase family protein
Phvul.007G048600	Cystein-rich RLK (RECEPTOR-like protein kinase) 29
Phvul.005G054300	Leucine-rich receptor-like protein kinase family protein
Phvul.007G048800	Cystein-rich RLK (RECEPTOR-like protein kinase) 29
Phvul.002G115900	Protein kinase superfamily protein
Phvul.007G030700	Protein kinase superfamily protein
Phvul.006G057900	Concanavalin A-like lectin protein kinase family protein
Phvul.001G019100	Mitogen-activated protein kinase kinase kinase 5
Phvul.008G174300	Protein kinase superfamily protein
Phvul.008G146000	Leucine-rich repeat receptor-like protein kinase family protein
Phvul.007G051400	Cystein-rich RLK (RECEPTOR-like protein kinase) 23
Phvul.008G175200	Protein kinase superfamily protein
Phvul.008G079800	Leucine-rich repeat protein kinase family protein
Phvul.010G088500	S-locus lectin protein kinase family protein
Phvul.007G064200	Protein kinase superfamily protein
Phvul.007G048500	cystein-rich RLK (RECEPTOR-like protein kinase) 29
Phvul.005G055000	Protein kinase superfamily protein
Phvul.006G169700	Integrin-linked protein kinase family
Phvul.011G150400	S-locus lectin protein kinase family protein
Phvul.002G207000	BRI1-associated receptor kinase
Phvul.006G066500	Calmodulin-binding receptor-like cytoplasmic kinase 1
Phvul.007G048900	Cystein-rich RLK (RECEPTOR-like protein kinase) 29
Phvul.002G018100	Mitogen-activated protein kinase kinase kinase 21
Phvul.010G118300	CBL-interacting protein kinase 9
Phvul.002G063600	Cystein-rich RLK (RECEPTOR-like protein kinase) 3
Phvul.002G023400	Protein kinase superfamily protein
Phvul.009G209200	Leucine-rich repeat protein kinase family protein
Phvul.008G225000	Leucine-rich repeat protein kinase family protein
Phvul.009G011400	Protein kinase superfamily protein
Phvul.006G108300	Leucine-rich repeat protein kinase family protein
Phvul.001G048400	Receptor serine/threonine kinase, putative
Phvul.002G000200	Receptor-like protein kinase 1
Phvul.002G002600	Protein kinase superfamily protein
Phvul.002G115800	Protein kinase superfamily protein
Phvul.008G029700	Malectin/receptor-like protein kinase family protein
Phvul.006G137300	Wall-associated kinase family protein
Phvul.003G226700	Protein kinase superfamily protein
Phvul.007G051000	Cystein-rich RLK (RECEPTOR-like protein kinase) 25
Phvul.007G030900	Protein kinase superfamily protein
Phvul.007G057000	With no lysine (K) kinase 1
Phvul.008G030000	Malectin/receptor-like protein kinase family protein

Transcript ID	Gene
Phvul.007G158400	Pyrophosphorylase 4
Phvul.002G276700	Diacylglycerol kinase 2
Phvul.007G137800	PEP1 receptor 1
Phvul.005G014900	Cystein-rich RLK (RECEPTOR-like protein kinase) 10
Phvul.006G200500	Concanavalin A-like lectin protein kinase family protein
Phvul.004G040000	Malectin/receptor-like protein kinase family protein
Phvul.011G064400	U-box domain-containing protein kinase family protein
Phvul.004G155600	Protein kinase superfamily protein
Phvul.010G099400	U-box domain-containing protein kinase family protein
Phvul.008G029900	Malectin/receptor-like protein kinase family protein
Phvul.002G220500	S-locus lectin protein kinase family protein
Phvul.010G057000	S-locus lectin protein kinase family protein
Phvul.006G084400	Cystein-rich RLK (RECEPTOR-like protein kinase) 3
Phvul.006G194600	Receptor kinase 3
Phvul.001G048200	Protein kinase superfamily protein
Phvul.010G057500	S-locus lectin protein kinase family protein
Phvul.006G190400	HAL2-like
Phvul.008G156400	Cystein-rich RLK (RECEPTOR-like protein kinase) 42
Phvul.002G318300	Protein kinase superfamily protein
Phvul.005G103200	Concanavalin A-like lectin protein kinase family protein
Phvul.008G233400	Protein kinase superfamily protein
Phvul.008G127000	S-locus lectin protein kinase family protein
Phvul.011G169300	Leucine-rich repeat transmembrane protein kinase
Phvul.009G239900	Protein kinase superfamily protein
Phvul.011G194700	Cystein-rich RLK (RECEPTOR-like protein kinase) 25
Phvul.007G210500	Protein kinase superfamily protein
Phvul.005G103400	Protein kinase superfamily protein
Phvul.002G063700	Cystein-rich RLK (RECEPTOR-like protein kinase) 2
Phvul.002G215000	Concanavalin A-like lectin protein kinase family protein
Phvul.001G135300	Calcium-dependent protein kinase 32
Phvul.004G173300	Root hair specific 16
Phvul.009G239800	Protein kinase family protein
Phvul.004G155300	Receptor serine/threonine kinase, putative
Phvul.008G081000	Malectin/receptor-like protein kinase family protein
Phvul.008G164500	Leucine-rich repeat protein kinase family protein
Phvul.004G164000	Proline extensin-like receptor kinase 1
Phvul.004G154800	Protein kinase superfamily protein
Phvul.009G244600	Leucine-rich repeat transmembrane protein kinase
Phvul.007G049400	Cystein-rich RLK (RECEPTOR-like protein kinase) 29
Phvul.007G049600	Cystein-rich RLK (RECEPTOR-like protein kinase) 29
Phvul.008G077500	Cystein-rich RLK (RECEPTOR-like protein kinase) 23/25
Phvul.001G096600	Protein kinase superfamily protein
Phvul.002G063900	Cystein-rich RLK (RECEPTOR-like protein kinase) 2
Phvul.002G152100	Leucine-rich repeat protein kinase family protein
Phvul.002G201400	Leucine-rich repeat protein kinase family protein
Phvul.002G017700	Protein kinase superfamily protein
Phvul.009G083300	Protein kinase superfamily protein
Phvul.002G108700	Calcium-dependent protein kinase 16
Phvul.005G085600	Protein kinase family protein
Phvul.006G150900	Protein kinase superfamily protein
Phvul.009G108500	Casein kinase I-like 3
Phvul.008G263900	Calmodulin-binding receptor-like cytoplasmic kinase 1
Phvul.011G119200	Concanavalin A-like lectin protein kinase family protein
Phvul.007G074000	Protein kinase superfamily protein

Transcript ID	Gene
Phvul.011G064000	Protein kinase superfamily protein
Phvul.001G243700	CRINKLY4 related 3
Phvul.001G265500	Leucine-rich repeat protein kinase family protein
Phvul.005G138900	Phototropin 1
Phvul.002G272100	Mitogen-activated protein kinase kinase kinase 3
Phvul.007G049000	Cystein-rich RLK (RECEPTOR-like protein kinase) 29
Phvul.005G162600	Leucine-rich repeat protein kinase family protein
Phvul.001G051100	S-domain-2 5
Phvul.008G264400	Leucine-rich repeat transmembrane protein kinase
Phvul.006G043700	Calmodulin-domain protein kinase 9
Phvul.007G031000	Protein kinase superfamily protein
Phvul.001G232300	Protein kinase superfamily protein
Phvul.001G049000	Protein kinase superfamily protein
Phvul.002G328300	MAP kinase 9
Phvul.011G167800	Leucine-rich repeat transmembrane protein kinase
Phvul.005G167700	cystein-rich RLK (RECEPTOR-like protein kinase) 10
Phvul.007G183000	ROP binding protein kinases 2
Phvul.004G154500	Protein kinase superfamily protein
Phvul.002G055600	Receptor-like protein kinase 1
Phvul.001G129300	6-phosphogluconate dehydrogenase family protein
Phvul.010G057400	Receptor-like protein kinase 4
Phvul.005G078400	Leucine-rich repeat transmembrane protein kinase
Phvul.002G215100	Leucine-rich repeat protein kinase family protein
Phvul.006G187700	Protein kinase superfamily protein
Phvul.009G133000	Protein kinase protein with adenine nucleotide alpha hydrolases-like domain
Phvul.001G048600	Protein kinase superfamily protein
Phvul.008G029600	Malectin/receptor-like protein kinase family protein
Phvul.002G242600	Receptor-like kinase in flowers 1
Phvul.003G120800	Protein kinase superfamily protein
Phvul.002G292400	MAP kinase 4
Phvul.005G135500	Protein kinase superfamily protein
Phvul.008G155300	Like SEX4 1
Phvul.001G090800	Protein kinase superfamily protein
Phvul.005G088700	S-locus lectin protein kinase family protein
Phvul.006G084800	Cystein-rich RLK (RECEPTOR-like protein kinase) 2
Phvul.001G130800	Wall associated kinase 5
Phvul.005G106800	STRUBBELIG-receptor family 3
Phvul.008G127100	S-locus lectin protein kinase family protein
Phvul.007G050700	Cystein-rich RLK (RECEPTOR-like protein kinase) 25
Phvul.001G048700	Protein kinase superfamily protein
Phvul.011G119100	Protein kinase superfamily protein
Phvul.010G165000	Protein kinase protein with adenine nucleotide alpha hydrolases-like domain
Phvul.002G049500	Cystein-rich RLK (RECEPTOR-like protein kinase) 25
Phvul.008G106600	Leucine-rich repeat receptor-like protein kinase family protein
Phvul.010G057600	Receptor-like protein kinase 1
Phvul.011G142200	Protein kinase superfamily protein
Phvul.005G079100	Leucine-rich repeat transmembrane protein kinase
Phvul.011G198200	Receptor serine/threonine kinase, putative
Phvul.002G227300	Protein kinase superfamily protein
Phvul.005G051000	Lectin protein kinase family protein
Phvul.003G187200	Leucine-rich repeat protein kinase family protein
Phvul.010G033800	Leucine-rich repeat transmembrane protein kinase family protein
Phvul.011G194400	Receptor kinase 1
Phvul.011G193900	Receptor kinase 2
Phvul.003G139200	Protein kinase superfamily protein
Phvul.001G049200	PR5-like receptor kinase
Phvul.005G164000	Leucine-rich repeat protein kinase family protein
Phvul.003G252900	Roline-rich extensin-like receptor kinase 4
Phvul.001G151100	Cyclin p1;1
Phvul.007G233900	Calcium dependent protein kinase 1
Phvul.003G269700	Protein kinase superfamily protein
Phvul.001G255800	Mitogen-activated protein kinase phosphatase 1
Phvul.010G018900	S-locus lectin protein kinase family protein
Phvul.011G008100	Leucine-rich repeat receptor-like protein kinase family protein
Phvul.010G070100	Protein kinase superfamily protein
Phvul.007G110300	CBL-interacting protein kinase 4
Phvul.001G076300	Protein kinase superfamily protein
Phvul.002G247900	Integrin-linked protein kinase family
Phvul.001G137300	Leucine-rich repeat receptor-like protein kinase family protein
Phvul.008G026700	Malectin/receptor-like protein kinase family protein
Phvul.008G105600	Leucine-rich repeat protein kinase family protein
Phvul.003G293600	Protein kinase superfamily protein
Phvul.008G084600	SOS3-interacting protein 1
Phvul.011G007200	Leucine-rich repeat protein kinase family protein
Phvul.008G081300	Protein kinase superfamily protein
Phvul.006G095700	ROP binding protein kinases 2
Phvul.008G154200	Leucine-rich repeat protein kinase family protein
Phvul.002G111100	Leucine-rich receptor-like protein kinase family protein
Phvul.001G134600	Leucine-rich repeat protein kinase family protein
Phvul.001G083300	Receptor-like protein kinase 2
Phvul.006G029000	Leucine-rich receptor-like protein kinase family protein
Phvul.002G223600	HAL2-like
Phvul.007G153400	Leucine-rich repeat transmembrane protein kinase family protein
Phvul.007G112100	Leucine-rich repeat protein kinase family protein
Phvul.009G082500	Leucine-rich receptor-like protein kinase family protein
Phvul.003G203100	Leucine-rich receptor-like protein kinase family protein
Phvul.005G185800	Leucine-rich repeat protein kinase family protein
Phvul.011G081800	Protein kinase family/ peptidoglycan-binding LysM domain-containing protein
Phvul.002G215200	Concanavalin A-like lectin protein kinase family protein
Phvul.004G147900	MAP kinase kinase 9
Phvul.006G217700	Protein kinase superfamily protein
Phvul.009G114400	Leucine-rich repeat transmembrane protein kinase family protein
Phvul.008G258400	Protein kinase superfamily protein
Phvul.002G184800	BRI1-like 2
Phvul.008G188700	Concanavalin A-like lectin protein kinase family protein
Phvul.007G279600	CBL-interacting protein kinase 7
Phvul.005G077600	Leucine-rich repeat transmembrane protein kinase
Phvul.008G086400	Leucine-rich receptor-like protein kinase family protein
Phvul.011G054300	Crinkly4
Phvul.007G103900	Protein kinase superfamily protein
Phvul.011G181900	Protein kinase superfamily protein
Phvul.010G086600	Protein kinase superfamily protein
Phvul.005G148800	AGC (cAMP/cGMP-dependent and protein kinase C) kinase family protein
Phvul.009G087500	CBL-interacting protein kinase 25
Phvul.011G035600	Protein kinase protein with adenine nucleotide alpha hydrolases-like domain
Phvul.010G044800	Cyclin p4;1
Phvul.006G149700	ATPase, F1 complex, gamma subunit protein
Phvul.008G124900	S-locus lectin protein kinase family protein
Phvul.011G194200	Cystein-rich RLK (RECEPTOR-like protein kinase) 25
Phvul.008G281500	Leucine-rich receptor-like protein kinase family protein

B

Transcript ID	Gene
Phvul.009G180700	Leucine-rich repeat protein kinase family protein
Phvul.006G006800	Cystein-rich RLK (RECEPTOR-like protein kinase) 25
Phvul.003G149200	Protein kinase superfamily protein
Phvul.001G073500	Casein kinase I-like 3
Phvul.008G265800	Protein kinase superfamily protein
Phvul.004G124100	Protein kinase superfamily protein
Phvul.003G137700	Leucine-rich repeat transmembrane protein kinase
Phvul.009G103100	Leucine-rich repeat protein kinase family protein
Phvul.002G267300	Protein kinase superfamily protein
Phvul.011G197500	Protein kinase superfamily protein
Phvul.001G069300	Protein kinase superfamily protein
Phvul.008G158100	Leucine-rich repeat protein kinase family protein
Phvul.003G162100	AGC (cAMP/cGMP-dependent and protein kinase C) kinase family protein
Phvul.007G051100	Cystein-rich RLK (RECEPTOR-like protein kinase) 25
Phvul.003G240400	Leucine-rich repeat protein kinase family protein
Phvul.011G070300	Phototropin 1
Phvul.009G131700	Wall associated kinase 3
Phvul.010G163200	Protein kinase superfamily protein
Phvul.010G111200	SPA (suppressor of phyA-105) protein family
Phvul.007G254000	Curculin-like (mannose-binding) lectin family protein
Phvul.011G197700	Receptor serine/threonine kinase, putative
Phvul.009G239600	Lectin protein kinase family protein
Phvul.006G198200	Leucine-rich receptor-like protein kinase family protein
Phvul.011G191900	Protein kinase superfamily protein
Phvul.006G105900	Phosphoenolpyruvate carboxylase kinase 1
Phvul.009G136200	Leucine-rich repeat transmembrane protein kinase family protein
Phvul.003G063700	Protein kinase family/ peptidoglycan-binding LysM domain protein
Phvul.011G193300	Cystein-rich RLK (RECEPTOR-like protein kinase) 10
Phvul.008G036300	Calcium/lipid-binding (CaLB) phosphatase
Phvul.008G016200	Adenylate cyclases
Phvul.006G175100	Leucine-rich repeat protein kinase family protein
Phvul.008G080500	Leucine-rich repeat protein kinase family protein
Phvul.008G211100	Chitin elicitor receptor kinase 1
Phvul.011G156200	S-locus lectin protein kinase family protein
Phvul.007G127200	Phosphoenolpyruvate carboxylase kinase 2
Phvul.002G146300	Protein kinase superfamily protein
Phvul.002G007800	HAESA-like 2
Phvul.009G102500	Kinase protein with adenine nucleotide alpha hydrolases-like domain
Phvul.009G130300	Kinase protein with adenine nucleotide alpha hydrolases-like domain
Phvul.008G058800	Wall associated kinase-like 2
Phvul.005G110400	S-locus lectin protein kinase family protein
Phvul.002G218800	Protein kinase superfamily protein
Phvul.011G196200	Cystein-rich RLK (RECEPTOR-like protein kinase) 10
Phvul.004G039900	Malectin/receptor-like protein kinase family protein
Phvul.008G212900	Leucine-rich repeat receptor-like protein kinase family protein
Phvul.007G077300	Protein kinase superfamily protein
Phvul.002G050100	NADH-Ubiquinone/plastoquinone (complex I) protein
Phvul.005G077900	Leucine-rich repeat transmembrane protein kinase

Transcript ID	Gene
Phvul.011G182300	Leucine-rich repeat receptor-like protein kinase family protein
Phvul.009G024200	Protein kinase superfamily protein
Phvul.010G018300	Leucine-rich repeat protein kinase family protein
Phvul.005G163500	Leucine-rich repeat protein kinase family protein
Phvul.011G153500	S-locus lectin protein kinase family protein
Phvul.008G144200	Cyclin p3;2
Phvul.002G179600	CBL-interacting protein kinase 3/9,Ca2+regulated ser-thr protein kinase
Phvul.008G056800	Wall associated kinase-like 2
Phvul.003G267000	Hercules receptor kinase 2
Phvul.004G126100	ERECTA-like 1
Phvul.005G088000	S-locus lectin protein kinase family protein
Phvul.007G050300	Cystein-rich RLK (RECEPTOR-like protein kinase) 25
Phvul.008G275000	Leucine-rich repeat protein kinase family protein
Phvul.011G175500	Cystein-rich RLK (RECEPTOR-like protein kinase) 12
Phvul.002G029200	Roline-rich extensin-like receptor kinase 4
Phvul.001G048500	PR5-like receptor kinase
Phvul.005G163100	Leucine-rich repeat protein kinase family protein
Phvul.011G152200	S-locus lectin protein kinase family protein
Phvul.004G040300	Malectin/receptor-like protein kinase family protein
Phvul.007G115500	Protein kinase superfamily protein
Phvul.005G113500	Cyclin p2;1
Phvul.001G146800	Protein kinase family protein
Phvul.007G188300	Malectin/receptor-like protein kinase family protein
Phvul.010G111000	Protein kinase family protein
Phvul.002G302700	Kinase protein with adenine nucleotide alpha hydrolases-like domain
Phvul.008G224900	Leucine-rich repeat protein kinase family protein
Phvul.001G047800	PR5-like receptor kinase
Phvul.005G043800	AGC kinase 1.7
Phvul.005G162500	Leucine-rich repeat protein kinase family protein
Phvul.003G082800	Protein kinase superfamily protein
Phvul.003G199900	Protein kinase superfamily protein
Phvul.007G112700	Mitogen-activated protein kinase kinase 3
Phvul.008G080000	Leucine-rich repeat protein kinase family protein
Phvul.005G165900	peptidoglycan-binding LysM domain-containing protein
Phvul.011G175300	Cystein-rich RLK (RECEPTOR-like protein kinase) 23, receptor kinase 1
Phvul.001G248700	SOS3-interacting protein 1
Phvul.008G159500	Leucine-rich repeat transmembrane protein kinase family protein
Phvul.009G131900	Wall-associated kinase 2
Phvul.006G071600	Leucine-rich repeat protein kinase family protein
Phvul.011G152300	S-locus lectin protein kinase family protein
Phvul.006G197000	BAK1-interacting receptor-like kinase 1
Phvul.008G030700	Malectin/receptor-like protein kinase family protein
Phvul.002G049300	Receptor kinase 1
Phvul.010G073000	Microtubule-associated proteins 70-3
Phvul.003G274800	Phototropin 2
Phvul.006G125300	Wall-associated kinase family protein
Phvul.005G110900	S-locus lectin protein kinase family protein

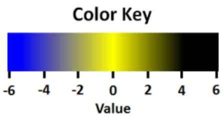

S10 Fig. DEGs of P metabolism genes during root symbioses. Expression profile of unique P metabolism genes in *P. vulgaris* roots colonized by (A) AMF and (B) rhizobia. Expression profile showing upregulated and downregulated DEGs obtained form GO analysis. Statistically significant DEGs were identified using an unpaired *t*-test (p<0.05), in symbiont treatment over controls (S3 Table). Fold-change values (over control) were used to plot heat maps. Color bar scale shows the fold-change range with red and green representing downregulation and upregulation, respectively.
