# Supplementary material for: Differentially expressed genes in mycorrhized and nodulated roots of common bean are associated with defense, cell wall architecture, N metabolism, and P metabolism
Source: PLoS One. 2017 Aug 3;12(8):e0182328. doi: 10.1371/journal.pone.0182328 (PMC5542541; doi:10.1371/journal.pone.0182328)
Supplement: S11 Fig — (PDF) [file pone.0182328.s011.pdf]

S11 Fig

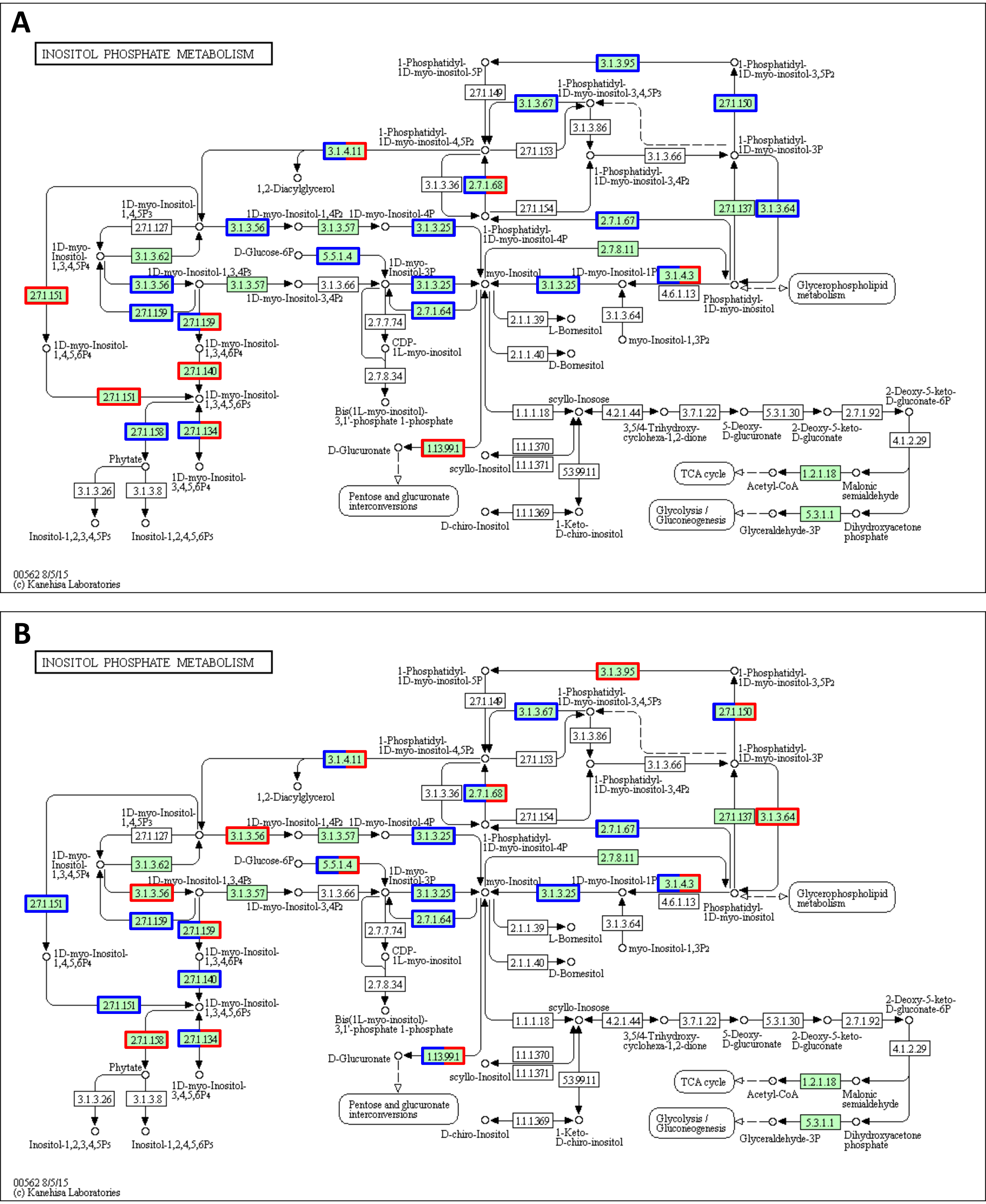

A
INOSITOL PHOSPHATE METABOLISM
1-Phosphatidyl-1D-myo-inositol-5P
3.1.3.95
1-Phosphatidyl-1D-myo-inositol-3,5P2
2.7.1.149
3.1.3.67
1-Phosphatidyl-1D-myo-inositol-3,4,5P3
2.7.1.150
3.1.3.86
1-Phosphatidyl-1D-myo-inositol-4,5P2
3.1.4.11
2.7.1.153
3.1.3.66
1-Phosphatidyl-1D-myo-inositol-3P
1,2-Diacylglycerol
3.1.3.36
2.7.1.68
1-Phosphatidyl-1D-myo-inositol-3,4P2
2.7.1.154
2.7.1.137
3.1.3.64
1D-myo-Inositol-1,4,5P3
2.7.1.127
3.1.3.56
1D-myo-Inositol-1,4P2
3.1.3.57
1D-myo-Inositol-4P
3.1.3.25
2.7.1.67
1-Phosphatidyl-1D-myo-inositol-4P
2.7.8.11
1D-myo-Inositol-1,3,4,5P4
3.1.3.62
D-Glucose-6P
5.5.1.4
1D-myo-Inositol-3P
myo-Inositol
1D-myo-Inositol-1P
3.1.4.3
Glycerophospholipid metabolism
2.7.1.151
1D-myo-Inositol-1,3,4P3
3.1.3.56
3.1.3.57
3.1.3.66
3.1.3.25
3.1.3.25
4.6.1.13
Phosphatidyl-1D-myo-inositol
1D-myo-Inositol-3,4P2
2.7.1.159
2.7.1.159
2.7.7.74
2.7.1.64
2.1.1.39
L-Bornesitol
3.1.3.64
myo-Inositol-1,3P2
1D-myo-Inositol-1,4,5,6P4
1D-myo-Inositol-1,3,4,6P4
CDP-1L-myo-inositol
2.1.1.40
D-Bornesitol
2.7.1.140
2.7.8.34
2.7.1.151
1D-myo-Inositol-1,3,4,5,6P5
Bis(1L-myo-inositol)-3,1'-phosphate 1-phosphate
scyllo-Inosose
1.1.1.18
4.2.1.44
3.7.1.22
5.3.1.30
2.7.1.92
2-Deoxy-5-keto-D-gluconate-6P
3,5/4-Trihydroxy-cyclohexa-1,2-dione
5-Deoxy-D-glucuronate
2-Deoxy-5-keto-D-gluconate
4.1.2.29
2.7.1.158
2.7.1.134
D-Glucuronate
1.13.99.1
1.1.1.370
scyllo-Inositol
1.1.1.371
5.3.99.11
Phytate
1D-myo-Inositol-3,4,5,6P4
TCA cycle
Acetyl-CoA
1.2.1.18
Malonic semialdehyde
3.1.3.26
3.1.3.8
Pentose and glucuronate interconversions
Glycolysis / Gluconeogenesis
5.3.1.1
1.1.1.369
D-chiro-Inositol
1-Keto-D-chiro-inositol
Glyceraldehyde-3P
Dihydroxyacetone phosphate
Inositol-1,2,3,4,5P5
Inositol-1,2,4,5,6P5
00562 8/5/15
(c) Kanehisa Laboratories
B
INOSITOL PHOSPHATE METABOLISM
1-Phosphatidyl-1D-myo-inositol-5P
3.1.3.95
1-Phosphatidyl-1D-myo-inositol-3,5P2
2.7.1.149
3.1.3.67
1-Phosphatidyl-1D-myo-inositol-3,4,5P3
2.7.1.150
3.1.3.86
1-Phosphatidyl-1D-myo-inositol-4,5P2
3.1.4.11
2.7.1.153
3.1.3.66
1-Phosphatidyl-1D-myo-inositol-3P
1,2-Diacylglycerol
3.1.3.36
2.7.1.68
1-Phosphatidyl-1D-myo-inositol-3,4P2
2.7.1.154
2.7.1.137
3.1.3.64
1D-myo-Inositol-1,4,5P3
2.7.1.127
3.1.3.56
1D-myo-Inositol-1,4P2
3.1.3.57
1D-myo-Inositol-4P
3.1.3.25
2.7.1.67
1-Phosphatidyl-1D-myo-inositol-4P
2.7.8.11
1D-myo-Inositol-1,3,4,5P4
3.1.3.62
D-Glucose-6P
5.5.1.4
1D-myo-Inositol-3P
myo-Inositol
1D-myo-Inositol-1P
3.1.4.3
Glycerophospholipid metabolism
2.7.1.151
1D-myo-Inositol-1,3,4P3
3.1.3.56
3.1.3.57
3.1.3.66
3.1.3.25
3.1.3.25
4.6.1.13
Phosphatidyl-1D-myo-inositol
1D-myo-Inositol-3,4P2
2.7.1.159
2.7.1.159
2.7.7.74
2.7.1.64
2.1.1.39
L-Bornesitol
3.1.3.64
myo-Inositol-1,3P2
1D-myo-Inositol-1,4,5,6P4
1D-myo-Inositol-1,3,4,6P4
CDP-1L-myo-inositol
2.1.1.40
D-Bornesitol
2.7.1.140
2.7.8.34
2.7.1.151
1D-myo-Inositol-1,3,4,5,6P5
Bis(1L-myo-inositol)-3,1'-phosphate 1-phosphate
scyllo-Inosose
1.1.1.18
4.2.1.44
3.7.1.22
5.3.1.30
2.7.1.92
2-Deoxy-5-keto-D-gluconate-6P
3,5/4-Trihydroxy-cyclohexa-1,2-dione
5-Deoxy-D-glucuronate
2-Deoxy-5-keto-D-gluconate
4.1.2.29
2.7.1.158
2.7.1.134
D-Glucuronate
1.13.99.1
1.1.1.370
scyllo-Inositol
1.1.1.371
5.3.99.11
Phytate
1D-myo-Inositol-3,4,5,6P4
TCA cycle
Acetyl-CoA
1.2.1.18
Malonic semialdehyde
3.1.3.26
3.1.3.8
Pentose and glucuronate interconversions
Glycolysis / Gluconeogenesis
5.3.1.1
1.1.1.369
D-chiro-Inositol
1-Keto-D-chiro-inositol
Glyceraldehyde-3P
Dihydroxyacetone phosphate
Inositol-1,2,3,4,5P5
Inositol-1,2,4,5,6P5
00562 8/5/15
(c) Kanehisa Laboratories

S11 Fig. Effect of root symbiosis on the inositol phosphate metabolism pathway in mycorrhized and nodulated *P. vulgaris* roots. The pathway model was obtained from KEGG Pathway [52]. Differential expression patterns of key genes and enzymes of inositol phosphate metabolism in *P. vulgaris* roots colonized by (A) AMF and (B) rhizobia. Blue and red borders surrounding EC identifiers represent genes that were upregulated and downregulated, respectively, relative to the controls. Borders with mixed colors indicate both upregulation and downregulation of different transcripts of the same gene.
