# Supplementary material for: Differentially expressed genes in mycorrhized and nodulated roots of common bean are associated with defense, cell wall architecture, N metabolism, and P metabolism
Source: PLoS One. 2017 Aug 3;12(8):e0182328. doi: 10.1371/journal.pone.0182328 (PMC5542541; doi:10.1371/journal.pone.0182328)
Supplement: S1 Table — (DOCX) [file pone.0182328.s012.docx]

**S1 Table. Primer sequences of *P. vulgaris* genes used to perform RT-qPCR analyses.**

| *P. vulgaris*  Locus Name | Gene | | Oligonucleotide sequence | Size of the amplicon (bp) | Reference |
| --- | --- | --- | --- | --- | --- |
| *Internal control genes* | | | | | |
| Phvul.004G075100 | PvElf1α | F | 5′-GGTCATTGGTCATGTCGACTCTGG-3′ | 146 | [33] |
|  |  | R | 5′-GCACCCAGGCATACTTGAATGACC-3′ |  |  |
| Phvul.001G133200 | PvIDE | F | 5′-GCAACCAACCTTTCATCAGC-3′ | 156 | [33] |
|  |  | R | 5′-AGAAATGCCTCAACCCTTTG-3′ |  |  |
| *Symbiont specific genes** | | | | |  |
| Phvul.004G007300 | PvPT-4 | F | 5′-GCGGTGACTAACATGTTAGGG-3′ | 137 | [34] |
|  |  | R | 5′-CCTGTGCCCTAGTATTGTTGG-3′ |  |  |
| Phvul.009G115800.1 | PvNIN | F | 5′-GGGGATTCAGAGATTTGCAG-3′ | 101 | [34] |
|  |  | R | 5′-AACCCACTCTTGAGCATCGT-3′ |  |  |
| *Validation of RNA-Seq data* | | | | | |
| Phvul.001G089900 | PvRAM1 | F | 5′-GATATTGACCAAACTACCCTAACCCCACCA-3′ | 147 | [17] |
|  |  | R | 5′-CTAATTGGTCAGCAAAAAAGGATTCCCAC-3′ |  |  |
| Phvul.007G233600 | PvRAM2 | F | 5′-GCTTTTTCCACCTTCCCAACCGT-3′ | 129 | [18] |
|  |  | R | 5′-AACCCCACCAGCTTCAAAGG-3′ |  |  |
| Phvul.001G111800 | PvERN1 | F | 5′-GGAGCTGTCTTTGATCGTTTTCC-3′ | 100 | [19] |
|  |  | R | 5′-CAAATTCAGAAAGCTCCAAGTCAGC-3′ |  |  |
| Phvul.002G064200 | PvENOD40 | F | 5′-AGTTTTGTTGGCAAGCATCC-3′ | 112 | [12] |
|  |  | R | 5′-TAAGCACAAGCAAACTGTTG-3′ |  |  |
| Phvul.008G098200 | ALC-INTERACTING 1 | F | 5′-AGTGGTGGCAAGGCTAATGG -3′ | 148 | - |
|  |  | R | 5′-GTGATGGTTGGCTTGGCTTG -3′ |  |  |
| Phvul.009G203400 | AGAMOUS-like 8 | F | 5′-GCACAATTTGAGCGACAAGC -3′ | 186 | - |
|  |  | R | 5′-TGTAGGACGAAGCATCCAAGG-3′ |  |  |
| Phvul.007G275700 | Hemopexin | F | 5′- TGATGCGTGTTTTGCTTCCC-3′ | 125 | - |
|  |  | R | 5′- GGATAGGCCTCACATCACCC -3′ |  |  |
| Phvul.002G218000 | Sulfite exporter TauE | F | 5′- GCAGGGATATTGGGTGGTGT-3′ | 186 | - |
|  |  | R | 5′- ATCCATGCCCAACAACAGGT-3′ |  |  |
| Phvul.007G141900 | Haemoglobin 2 | F | 5′-GCACCAGCAGCAAAGAACTTGT-3′ | 159 | - |
|  |  | R | 5′-ACCAAGTGCAGCATCAGCCACC-3′ |  |  |
| Phvul.001G112800 | Spermidine HOC | F | 5′-ACCCACGTCCCCATGATCTA -3′ | 177 | - |
|  |  | R | 5′-GGTGGCGTTGCAATCAAGTT-3′ |  |  |
| Phvul.002G058900 | C2H2 zinc finger like | F | 5′-AAGTTGAGGAGAGTTCGCCG-3′ | 168 | - |
|  |  | R | 5′- CAGTGCTTGACCAGACCCAA-3′ |  |  |
| Phvul.007G034700 | MATE efflux | F | 5′-TGGAGCATTGGCAGTTAGCA-3′ | 171 | - |
|  |  | R | 5′-AGGCAAACCATTACTGCCGA -3′ |  |  |

*For validating absence of mycorrhiza and rhizobia cross contamination.
