# Supplementary material for: Differentially expressed genes in mycorrhized and nodulated roots of common bean are associated with defense, cell wall architecture, N metabolism, and P metabolism
Source: PLoS One. 2017 Aug 3;12(8):e0182328. doi: 10.1371/journal.pone.0182328 (PMC5542541; doi:10.1371/journal.pone.0182328)
Supplement: S2 Table — (DOC) [file pone.0182328.s013.doc]

**S2 Table. Summary of the number of Ion Proton sequencer reads and BLASTX hits to the *P. vulgaris* genome database.**

| Root sample | Total number of reads | High quality reads | Mean read length | Longest alignment |
| --- | --- | --- | --- | --- |
| **Control ^#^** | 37 903 671 | 34 871 377 | 186 bp | 326 bp |
| **Mycorrhized** | 38 894 101 | 36 560 454 | 209 bp | 334 bp |
| **Nodulated** | 37 999 022 | 35 932 910 | 215 bp | 341 bp |

# Uninoculated roots. Data presented are the average of two biological replicates.
