# Supplementary material for: Differentially expressed genes in mycorrhized and nodulated roots of common bean are associated with defense, cell wall architecture, N metabolism, and P metabolism
Source: PLoS One. 2017 Aug 3;12(8):e0182328. doi: 10.1371/journal.pone.0182328 (PMC5542541; doi:10.1371/journal.pone.0182328)
Supplement: S5 Table — (DOCX) [file pone.0182328.s016.docx]

**S5 Table. Total number of transcription factors of different families that are differentially expressed in mycorrhized and nodulated *P. vulgaris* roots.**

|  | **No. of genes** | **Gene ID** | **Gene description** | **Expression** | |
| --- | --- | --- | --- | --- | --- |
|  |  |  |  | **AMF** | **RNS** |
| Overlapping AMF and RNS genes | 4 | AP2 domain family genes | Phvul.003G069000 | -3.2740410 | -3.2740411 |
|  |  |  | Phvul.007G193700 | -3.2487965 | -3.5704384 |
|  |  |  | Phvul.009G013200 | 3.2366319 | -2.0334291 |
|  |  |  | Phvul.009G137900 | 7.0703216 | -2.8714461 |
|  | 9 | Ethylene-responsive transcription factor family genes | Phvul.004G092100 | 6.7049394 | 2.1256323 |
|  |  |  | Phvul.010G050700 | 4.4971270 | 4.4988923 |
|  |  |  | Phvul.010G050600 | 2.7940593 | 3.7000290 |
|  |  |  | Phvul.010G114900 | 2.6704974 | 2.6885410 |
|  |  |  | Phvul.011G118600 | -2.0048337 | -3.6054132 |
|  |  |  | Phvul.001G160400 | 3.5115314 | -2.666699 |
|  |  |  | Phvul.007G222500 | 8.7526600 | 4.7880654 |
|  |  |  | Phvul.010G050500 | 4.0287905 | 4.3753643 |
|  |  |  | Phvul.010G159500 | -2.900313 | 5.0558080 |
|  | 4 | Homeobox transcription family genes | Phvul.009G077100 | 3.0002189 | -2.2964635 |
|  |  |  | Phvul.011G082200 | 4.2911277 | 4.9378130 |
|  |  |  | Phvul.002G137000 | 4.0132694 | 3.1189995 |
|  |  |  | Phvul.001G143500 | -5.0828824 | -2.4995394 |
|  | 4 | MADS-box genes | Phvul.007G065100 | 2.6452260 | -4.6391253 |
|  |  |  | Phvul.007G047800 | 2.0977557 | -2.9656422 |
|  |  |  | Phvul.004G042500 | 3.7818174 | -3.6239400 |
|  |  |  | Phvul.002G212500 | 2.1308768 | -2.7104604 |
| Unique AMF genes | 15 | AP2 domain family genes | Phvul.002G035100 | 5.1242140 | - |
|  |  |  | Phvul.007G193400 | 4.1978817 | - |
|  |  |  | Phvul.001G187100 | 4.0133467 | - |
|  |  |  | Phvul.009G084400 | 3.8807173 | - |
|  |  |  | Phvul.007G272900 | 3.3787317 | - |
|  |  |  | Phvul.001G044500 | 3.1745625 | - |
|  |  |  | Phvul.009G123300 | 2.6663713 | - |
|  |  |  | Phvul.007G135900 | 2.2715425 | - |
|  |  |  | Phvul.002G055700 | 2.236887 | - |
|  |  |  | Phvul.008G098900 | 2.1666641 | - |
|  |  |  | Phvul.002G035900 | 2.013405 | - |
|  |  |  | Phvul.003G222600 | -3.5646667 | - |
|  |  |  | Phvul.001G160100 | -3.3112416 | - |
|  |  |  | Phvul.007G135300 | -2.7376204 | - |
|  |  |  | Phvul.002G254500 | -2.206746 | - |
|  | 15 | Ethylene-responsive transcription factor family genes | Phvul.002G267800 | 8.495295 | - |
|  |  |  | Phvul.003G212700 | 5.450528 | - |
|  |  |  | Phvul.003G223600 | 5.3328485 | - |
|  |  |  | Phvul.002G149500 | 4.363578 | - |
|  |  |  | Phvul.006G179700 | 3.6143105 | - |
|  |  |  | Phvul.003G111800 | 3.6070633 | - |
|  |  |  | Phvul.006G179800 | 3.3559747 | - |
|  |  |  | Phvul.002G036000 | 3.1304522 | - |
|  |  |  | Phvul.003G292400 | 3.024023 | - |
|  |  |  | Phvul.001G084000 | 2.782017 | - |
|  |  |  | Phvul.002G163700 | 2.6276064 | - |
|  |  |  | Phvul.007G102800 | 2.3079996 | - |
|  |  |  | Phvul.007G241600 | -2.8582497 | - |
|  |  |  | Phvul.008G019600 | -2.10607 | - |
|  |  |  | Phvul.003G107900 | -2.0194206 | - |
|  | 8 | Homeobox transcription family genes | Phvul.004G120500 | 3.9525194 | - |
|  |  |  | Phvul.010G148700 | 3.2913985 | - |
|  |  |  | Phvul.009G190200 | 3.0279212 | - |
|  |  |  | Phvul.002G187900 | 2.361031 | - |
|  |  |  | Phvul.007G224800 | 2.321642 | - |
|  |  |  | Phvul.008G042200 | -2.8474123 | - |
|  |  |  | Phvul.002G064100 | -2.437333 | - |
|  |  |  | Phvul.001G034200 | -2.0313416 | - |
|  | 5 | MADS-box genes | Phvul.009G203400 | 7.783833 | - |
|  |  |  | Phvul.004G042400 | 3.3653555 | - |
|  |  |  | Phvul.002G215500 | 2.305751 | - |
|  |  |  | Phvul.009G037300 | 2.2242994 | - |
|  |  |  | Phvul.008G027900 | 2.1148233 | - |
| Unique RNS genes | 4 | AP2 domain family genes | Phvul.007G086600 | - | 3.4688823 |
|  |  |  | Phvul.006G106100 | - | -2.7558951 |
|  |  |  | Phvul.008G043500 | - | 4.451249 |
|  |  |  | Phvul.011G091400 | - | -2.1038184 |
|  | 6 | Ethylene-responsive transcription factor family genes | Phvul.009G196900 | - | 6.0636253 |
|  |  |  | Phvul.001G111800 | - | 4.1339335 |
|  |  |  | Phvul.010G050800 | - | 2.654746 |
|  |  |  | Phvul.001G160200 | - | -2.7989638 |
|  |  |  | Phvul.005G105200 | - | -2.3647242 |
|  |  |  | Phvul.007G272800 | - | -2.0807347 |
|  | 11 | Homeobox transcription family genes | Phvul.011G064900 | - | 6.275299 |
|  |  |  | Phvul.002G048200 | - | 4.373028 |
|  |  |  | Phvul.008G045100 | - | 4.137895 |
|  |  |  | Phvul.006G013200 | - | 3.7770042 |
|  |  |  | Phvul.006G145800 | - | 3.3395994 |
|  |  |  | Phvul.002G027200 | - | 2.3422623 |
|  |  |  | Phvul.002G056300 | - | 2.0109873 |
|  |  |  | Phvul.005G168900 | - | -3.460281 |
|  |  |  | Phvul.009G176700 | - | -2.5514436 |
|  |  |  | Phvul.001G106700 | - | -2.5303586 |
|  |  |  | Phvul.003G251800 | - | -2.2729135 |
|  | 7 | MADS-box genes | Phvul.007G048000 | - | 6.263799 |
|  |  |  | Phvul.003G182700 | - | 3.5545614 |
|  |  |  | Phvul.002G143900 | - | -4.5530076 |
|  |  |  | Phvul.010G088100 | - | -3.3983357 |
|  |  |  | Phvul.006G202200 | - | -2.8736608 |
|  |  |  | Phvul.003G182800 | - | -2.6194715 |
|  |  |  | Phvul.002G112200 | - | -2.0511823 |

These data were obtained from DEGs of N metabolism.
